# Study on the Multitarget Mechanism of Sanmiao Pill on Gouty Arthritis Based on Network Pharmacology

**DOI:** 10.1155/2020/9873739

**Published:** 2020-08-04

**Authors:** Huiqin Qian, Qianqian Jin, Yichen Liu, Ning Wang, Yuru Chu, Bingbing Liu, Yan Liu, Wanli Jiang, Yong Song

**Affiliations:** ^1^College of Pharmacy, Sanquan College of Xinxiang Medical University, Xinxiang 453000, China; ^2^Otolaryngological Department, Puyang Oilfield General Hospital, Puyang 457000, China

## Abstract

Sanmiao pill (SMP), a Chinese traditional formula, had been used to treat gouty arthritis (GA). However, the active compounds and underlying mechanism remained unclear. Hence, network pharmacology and molecular docking were utilized to explore bioactive compounds and potential mechanism of action of SMP in treating GA. In the study, the compounds of SMP, corresponding targets, and GA-related targets were mined from various pharmacological databases. Then, herb-compound-target, compound-target, PPI, and target-pathway networks were constructed. Ultimately, molecular docking was carried out to verify the predicted results. The results indicated that 47 active compounds, 338 targets, and 144 disease targets were collected. Network analysis implied that *Phellodendron chinense* Schneid. played a vital role in the whole formula. Moreover, 7 compounds (quercetin, kaempferol, wogonin, rutaecarpine, baicalein, beta-sitosterol, and stigmasterol) and 4 targets (NFKB1, RELA, MAPK1, and TNF) might be the kernel compounds and targets of SMP against GA. According to GOBP and KEGG pathway enrichment analysis and target-pathway network, SMP might exert a therapeutic role in GA by regulating numerous biological processes and pathways, including lipopolysaccharide-mediated signaling pathway, positive regulation of transcription, Toll-like receptor signaling pathway, JAK-STAT signaling pathway, NOD-like receptor signaling pathway, and MAPK signaling pathway. The results of molecular docking showcased that 11 pairs of compound with targets had tight binding strength. Thereinto, 4 compounds of MAPK1 and 5 compounds of NFKB1 possessed a better combination, suggesting that MAPK1 and NFKB1 might be considered as therapeutic targets in treatment of GA. This study verified that SMP had synergistic effect on GA by multicomponents, multitargets, and multipathways.

## 1. Introduction

Gouty arthritis (GA) was a common inflammatory arthritis, which affected at least 1% of the population in developed countries and increased obviously over the last few decades [1]. Due to reduction of uric acid excretion occur and/or purine metabolism disorders, the deposition of monosodium urate (MSU) crystals in synovial fluid and other tissues in the presence of elevated urate concentrations gave rise to GA [2]. The main symptoms of GA included severe pain, swelling, and tenderness resulting in difficulty moving the affected joint(s) [3], which significantly lowered the quality of life and caused a substantial economic burden [4]. In addition, GA could also bring in a series of comorbidities including obesity, hypertension, type 2 diabetes mellitus, coronary heart disease, metabolic syndrome, and renal disease [5, 6]. The remedies for GA were mainly nonsteroidal anti-inflammatory drugs, colchicine, or glucocorticoids which led to serious side reactions [1, 7]. Accordingly, hunting for therapeutic drugs in treatment of GA was urgently required.

Recently, traditional Chinese medicine (TCM) had been regarded as alternative therapies or dietary supplements for treatment of GA. Sanmiao pill (SMP), a basic traditional prescription for treatment of GA since the Ming Dynasty, was composed of three TCMs: *Phellodendron chinense* Schneid. (Huangbo, HB), *Achyranthes bidentata* Blume. (Niuxi, NX), and *Atractylodes lancea* (Thunb.) DC. (Cangzhu, CZ) [8]. One study had reported that *Achyranthes bidentata* Blume. accumulated berberine in the knee joint and enhanced the anti-inflammatory effect under its lower-guiding drug in SMP [9]. In addition, SMP intervened in the expression of matrix metalloproteinases (MMPs)-3 and aggrecanases (ADAMTSs)-4 and enhanced the expression of GA-reduced tissue inhibitors (metalloproteinases (TIMPs)-1 and -3), resulting in inhibition of cartilage matrix degradation effectively in GA [10]. Although recent pharmacological studies had demonstrated that SMP might exert protective effect on GA, the underlying molecular mechanism of SMP acting on GA had not been fully elucidated.

**Figure 1:**
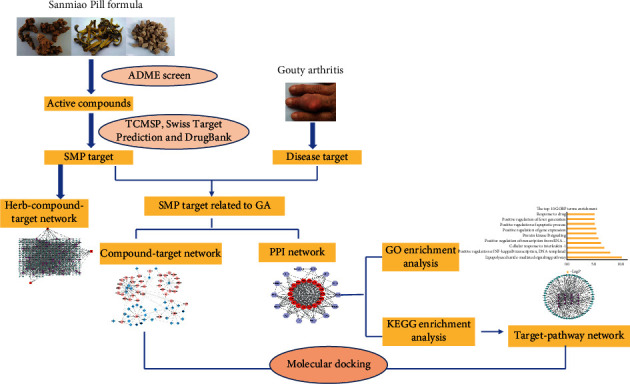
The flowchart of network pharmacology approach.

Network pharmacology was emerging discipline which integrated network biology and polypharmacology [11]. Recently, a growing number of network pharmacology had been applied to comprehensively and systematically elucidate active compounds and potential mechanisms of action of TCM formulas by integrating multiple networks between compounds, targets, diseases, and pathways [12]. It provided a new sight into evaluating the efficacy mechanisms of drugs. The study integrated network pharmacology and molecular docking to firstly uncover underlying mechanism of SMP in treatment of GA. The detailed flowchart in the study is depicted in Figure 1.

## 2. Materials and Methods

### 2.1. Identification of Bioactive SMP Compounds

The compounds in SMP formula were harvested from the Traditional Chinese Medicine Systems Pharmacology Database (TCMSP, http://ibts.hkbu.edu.hk/LSP/tcmsp.php). Then, all compounds in SMP were subjected to ADME screening, which was evaluated by the possibility of each compounds in SMP as drug and carried out by the TCMSP database. In the integrative ADME model, oral bioavailability (OB) and drug-likeness (DL) were significantly pharmacokinetic parameters and regarded as screening criteria. Therefore, active compounds were remained which were satisfactory with criteria of OB ≥ 30% and DL ≥ 0.18 for further study [13]. The simplified molecular-input line-entry specification (SMILES) of active compounds was ultimately downloaded from PubChem (https://pubchem.ncbi.nlm.nih.gov/) and OSRA (https://cactus.nci.nih.gov/osra/) databases.

### 2.2. Prediction Targets of SMP

SMP targets were retrieved from three public online databases, namely, TCMSP, Swiss TargetPrediction, and DrugBank databases. Thereinto, Swiss TargetPrediction and DrugBank databases predicted targets per active compound based on the similarity of chemical structure. Therefore, in order to ensure the credibility of the targets, the targets with probability ≥0.5 were retained from the Swiss TargetPrediction database. Meanwhile, the targets which met the criteria of similar drugs approved by FDA with similarity threshold ≥0.7 were selected from the DrugBank database. In addition, the corresponding targets of active ingredients were supplemented through the TCMSP database. Taken together, the SMP targets were imported from UniProt (http://www.uniprot.org/) by restricting the species to “*Homo sapiens*” to standardize the gene name for the following data processing.

### 2.3. Construction of Herb-Compound-Target Network

An herb-compound-target network was established by Cytoscape (3.6.0) software, so as to explore the relationship between herbs in SMP formula, compounds, and corresponding targets. Degree, one of topological parameters, which was calculated by network analysis, was applied to preliminarily identify the mainstream herb, compound, and target.

### 2.4. SMP Targets Related to GA

The GA-relevant targets were collected from GeneCards (https://www.genecards.org/) and DisGeNet (http://www.disgenet.org/search) with the keywords “gouty arthritis” and “gouty.” The standardized gene names were obtained from UniProt (http://www.uniprot.org/) as well. The compound-related targets and GA-related targets were, respectively, put into the Venn software which was from the OmicShare platform (https://www.omicshare.com/). Ultimately, we could acquire SMP-related targets on GA for the following study.

### 2.5. Construction of Compound-Target Network

To accurately elicit interactions between active compounds and its corresponding GA-related targets of SMP, we constructed a compound-target network. Furthermore, topological parameters were applied eventually to screen key active compounds and GA-related targets of SMP.

### 2.6. Construction of Protein-Protein Interaction (PPI) Network

GA-related targets of SMP were then processed by the String database (https://string-db.org/cgi/input.pl) to acquire protein-protein interactions (PPIs). “Organism” was set option to “*Homo sapiens*,” and only PPI with a combined score more than 0.4 was selected in the specific operation process. The PPI network of GA-related targets of SMP was built through Cytoscape (3.6.0) software. As previously reported [14], we selected the targets whose degree value was greater than the average of all the other nodes in the network as hub targets of SMP-related GA.

### 2.7. GO and KEGG Pathway Analysis

In order to systematically analyze Gene Ontology (GOBP) and the KEGG pathway of SMP targets related to GA, we imported hub targets into DAVID Bioinformatics Resources 6.8 (https://david.ncifcrf.gov/). The GOBP terms and KEGG pathways with *P* ≤ 0.05 were screened for further analysis.

### 2.8. Construction of Target-Pathway Network

The target-pathway network was built by the linkage with hub targets and KEGG pathways so as to detect the mechanism of SMP acting on GA. Subsequently, we selected the pathway with degree more than or equal to median value and associated with GA as a major pathway for further study.

### 2.9. Molecular Docking Verification

To validate the significant compounds and targets which were selected from analysis of compound-target and target-pathway networks, the AutoDock tool was applied to perform a molecular docking. First of all, we prepared 3D structure of targets and compounds which were mined from PDB (https://www.rcsb.org/) and PubChem (https://pubchem.ncbi.nlm.nih.gov/) databases before performing the docking progress, respectively. Then, the target proteins were imported into the AutoDock tool for a series of processes including removing H_2_O, adding hydrogen atoms, and calculating charge and atom type. The grid box was the docking area of the protein and compound which covered as much the protein and ligand as possible. Molecular docking employed the Lamarckian genetic algorithm (LGA). Meanwhile, we ignored all bond rotations of ligand. Ultimately, the docking binding energy of every compound-protein was calculated via the AutoDock tool to assess binding interactions.

## 3. Results and Discussion

### 3.1. Selection of Compounds in Herbal Medicines Using ADME Evaluation

364 compounds of the whole formula were mined from the TCMSP database in total, comprising 49 in CZ, 139 in HB, and 176 in NX. Among which, 19 compounds were excluded owing to duplicate (Supplementary Table 1). 56 active compounds of SMP were initially selected which met screening criteria of OB ≥ 30% and DL ≥ 0.18. However, owing to 9 bioactive compounds had no corresponding targets for the subsequent putative targets and thus excluded, we ultimately obtained 47 active compounds (Table 1).

### 3.2. Active Compounds Related to Target

47 compounds hit 338 targets, including 88 in CZ, 306 in HB, and 313 in NX obtained from TCMSP, Swiss TargetPrediction, and DrugBank databases. Details of compound targets were prescribed in Supplementary Table 2.

### 3.3. Herb-Compound-Target Network

**Figure 2:**
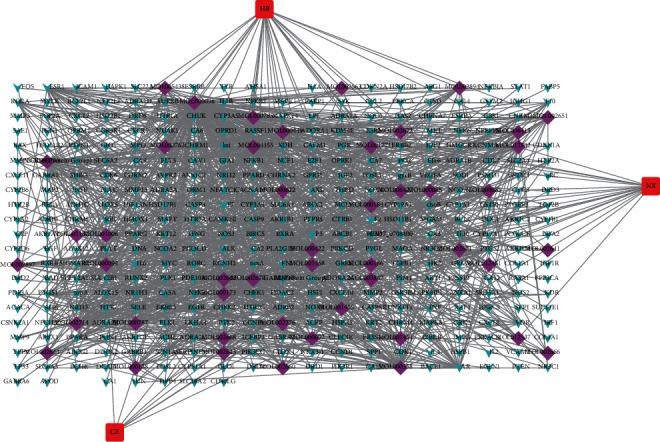
Network of herbs in SMP, compounds, and corresponding targets. The blue V's depict the targets, purple diamonds delineate the compounds, and red rectangles portray the herbs (CZ, HB, and NX) in SMP.

The herb-compound-target network was built so as to preliminarily understand the interactions between herbs, compounds, and targets. The network embodied 388 nodes (3 herb nodes, 47 compound nodes, and 338 compound target nodes) and 1255 edges (Figure 2). Degree, one of topological parameters, which referred to the number of nodes connected directly with it, was applied to assess the importance of herbs and compounds in the network. Degree values were calculated by network analysis (Supplementary Table 3). The degree values of HB, CZ, and NX were, respectively, 29, 8, and 9, suggesting that one herb was capable to correlated closely numerous active compounds. Meanwhile, HB displayed the highest degree value, indicating that HB potentially played a crucial role in SMP. A previous study had vindicated the anti-inflammation effect of HB in various animal inflammatory models by adjusting release of nitric oxide (NO), inducing nitric oxide synthase (iNOS) production, attenuating the release of tumor necrosis factor-*α* (TNF-*α*) and interleukin 1*β* (IL-1*β*) [15]. Moreover, 47 active compounds had at least linkage with two targets, showcasing that a multitude of targets portioned mutual compounds. Besides, the mean degree value of targets was 3.55, respectively, signifying that each targets correlated with an average of 3.55 compounds, which further confirmed that a compound could not only affect one target, but it also could act on multiple targets and exert potential synergistic effects.

### 3.4. SMP Targets Associated with GA

**Figure 3:**
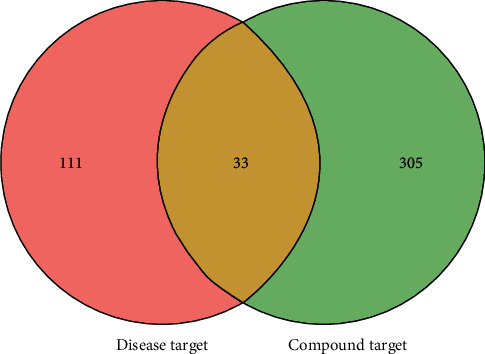
Venn diagram of compound targets and disease targets.

We obtained a total of 144 disease targets from the two pharmacology databases, including 138 targets in the GeneCards database, 37 targets in the DisGeNet database, and 31 redundancies which were excluded (Supplementary Table 4).  Figure 3 displays that 33 different GA-related targets were acquired by matching 144 disease targets to 338 compound targets.

### 3.5. Compound-Target Network

**Figure 4:**
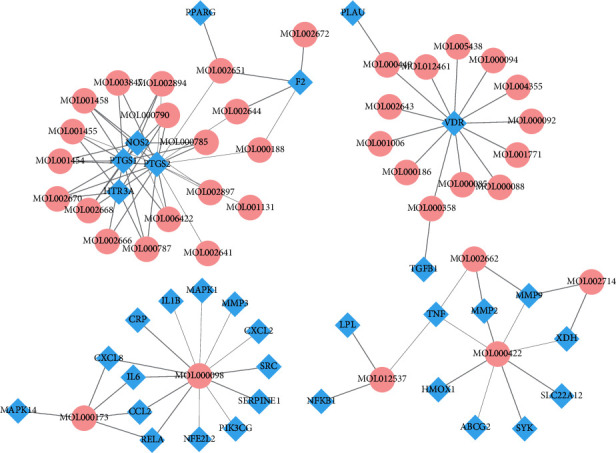
Compound-target network (blue diamonds represent active compounds. Pink ellipses delineate SMP targets related to GA).

To disclose the intimate interactions of compounds and GA-related targets of SMP, the compound-target (C-T) network was constructed. C-T network embodied 71 nodes (38 compounds and 33 SMP targets related to GA) and 130 edges (Figure 4). Based on the network analysis, average degree value of each compound was 3.42 (Supplementary Table 5). Nodes with degrees only greater than the average degree could be significant compounds. Quercetin (degree = 27), kaempferol (degree = 14), wogonin (degree = 10), rutaecarpine (degree = 6), baicalein (degree = 5), beta-sitosterol (degree = 4), and stigmasterol (degree = 4) were identified as kernel bioactive compounds of SMP for the subsequence molecular docking, owing to hitting overall more than 4 targets. A previous study had demonstrated that quercetin exerted anti-inflammatory and analgesic outcomes on an animal model of MSU-induced acute gout via intervening hyperalgesia, recruiting leukocyte, producing superoxide anion, and activating inflammasome. Meanwhile, quercetin showed analgesic and anti-inflammatory effect in treating acute gouty [16]. Kaempferol exhibited therapeutic effect on rheumatoid arthritis and intervened the migration and invasion of fibroblast-like synoviocytes by obstructing MAPK pathway activation [17]. Wogonin could restrain effectively extrinsic and intrinsic apoptotic pathways and induce antiapoptotic proteins in treatment of osteoarthritis chondrocytes [18]. Additionally, the average degree value of per target was 3.94. 10 targets (PTGS2, PTGS1, VDR, NOS2, F2, HTR3A, TNF, MMP9, PPARG, and RELA) were regulated by more than or equal to 4 bioactive compounds, which might play a critical role in treating GA.

### 3.6. PPI Network

**Figure 5:**
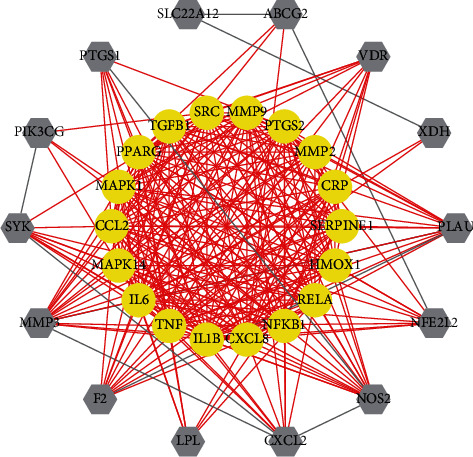
PPI network. The light purple hexagons represent the SMP targets related to GA and yellow ellipses delineate the hub target of SMP targets related to GA.

To shed light on the hub GA-relevant targets of SMP for the following GO and KEGG pathway enrichment, the PPI network was visualized by Cytoscape (3.6.0) software with 32 nodes and 264 edges based on the protein-protein interactions from the String database (Figure 5). The degree value of each target was calculated. The targets with degree value greater than average (16.5) were designated as the hub GA-associated targets of SMP (Supplementary Table 6). We eventually selected 18 hub targets, which were potential therapeutic effects on GA, for instance, tumor necrosis factor (TNF), interleukin-6 (IL6), interleukin-8 (CXCL8), C-C motif chemokine 2 (CCL2), interleukin-1 beta (IL1B), tyrosine-protein kinase SRC (SRC), prostaglandin-endoperoxide synthase 2 (PTGS2), mitogen-activated protein kinase 1 (MAPK1), peroxisome proliferator-activated receptor gamma (PPARG), transforming growth factor beta-1 (TGFB1), matrix metalloproteinase-9 (MMP9), transcription factor p65 (RELA), mitogen-activated protein kinase 14 (MAPK14), plasminogen activator inhibitor 1 (SERPINE1), heme oxygenase 1 (HMOX1), nuclear factor NF-kappa-B p105 subunit (NFKB1), 72 kDa type IV collagenase (MMP2), and C-reactive protein (CRP).

It was reported that the pathogenesis of GA was closely related to the NLRP3 inflammasome and inflammatory cytokines [19]. MSU crystals could be recognized by pattern recognition receptors and bind to NALP3 inflammasome. In turn, that leaded to the increasing number of mononuclear macrophages to engulf MSU crystals. Eventually, histamines and chemokines were released, resulting in inflammatory reactions such as local vasodilation and leukocyte accumulation. In addition, inflammatory factors were produced and secreted, such as TNF, IL1B, IL6, CXCL8, and CCL2, which aggravated the inflammatory reaction of MSU crystal deposition area and further amplified the inflammatory reaction of GA [20]. MAPK1 and MAPK14, the serine-threonine protein kinase family, modulated multiple cellular activities, such as cell proliferation, apoptosis, inflammation, and innate immunity. The MAPK signaling pathway was significant regulators of inflammatory cytokine which was activated by MAPK phosphorylation [21]. Oxidative stress played an important role in NLRP3 inflammasome activation. When oxidative stress was suppressed, the generation and secretion of IL-1*β* induced by NLRP3 activation would be blocked, whereas HMOX1 modulated the balance of redox signaling. Consequently, HMOX1 also played an indirect role in the development of GA [22]. MMP2 and MMP9 were the members of the matrix metalloproteinase (MMP) family, which increased cartilage degradation, further resulting in the development of GA [23]. SERPINE1, one of the serine protease inhibitors, played a pathophysiological role in the synovial fluid of gouty arthritis [24]. CRP, an acute reactant protein, had a primitive defense function against the exogenous stimulus, which was significant part of inflammation [25]. COX2 was encoded by the PTGS2 gene, which participated in pathological processes such as inflammation and fever [26]. RELA and NFKB1, the transcription factor protein family, were the central mediator of the inflammatory process [27]. In addition, SRC, PPARG, and TGFB1 also regulated the inflammatory response [28–30].

### 3.7. GO and KEGG Pathway Analysis

**Figure 6:**
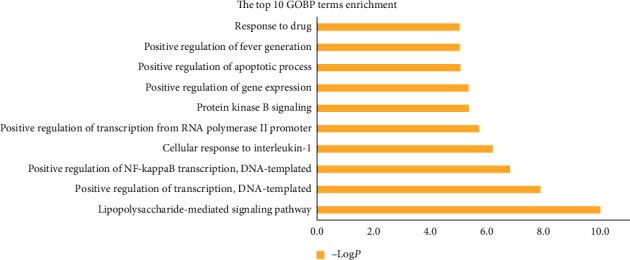
GOBP enrichment analysis of hub targets (the *y*-axis represents top 10 BP terms, and the *x*-axis represents the *P* values.

To clarify the molecular mechanisms of GA-related targets of SMP, GOBP and KEGG pathway enrichment of 18 hub GA-related targets was performed. A total of 44 GOBP terms were enriched with *P* ≤ 0.05 (Supplementary Table 7). The top significant GOBP terms are shown in Figure 6. The results indicated that GA-related targets of SMP could participate in a majority of biological process in treatment of GA, such as lipopolysaccharide-mediated signaling pathway, positive regulation of transcription, DNA-templated, positive regulation of NF-kappa B transcription factor activity, cellular response to interleukin-1, positive regulation of transcription from RNA polymerase II promoter, protein kinase B signaling, positive regulation of gene expression, positive regulation of apoptotic process, positive regulation of fever generation, and response to drug.

According to KEGG pathway enrichment analysis, 17 of 18 SMP targets related to GA enriched 57 pathways with *P* ≤ 0.05 (Supplementary Table 8), which were divided into six categories: immunity, inflammation, endocrine system, nervous system, disease and cell proliferation, and differentiation and migration. Thereinto, 32 disease-related pathways not only included infectious diseases such as Chagas disease, influenza A, hepatitis B, and malaria but also included inflammatory processes of chronic diseases such as rheumatoid arthritis, insulin resistance, and cancer. In addition, 2 inflammation-related pathways were cytokine-cytokine receptor interaction and adipocytokine signaling pathway. 7 immunity-related pathways included the T-cell receptor signaling pathway, cytosolic DNA-sensing pathway, hematopoietic cell lineage, etc.. 8 pathways associated with inflammation and immunity were VEGF signaling pathway, NF-kappa B signaling pathway, TNF signaling pathway, NOD-like receptor signaling pathway, Toll-like receptor signaling pathway, etc. Ultimately, pathways associated with endocrine system, nervous system, and cell proliferation, differentiation, and migration were, respectively, 3, 1, and 4. This suggested that SMP could treat GA by acting on multiple pathways.

### 3.8. Construction of Target-Pathway Network

**Figure 7:**
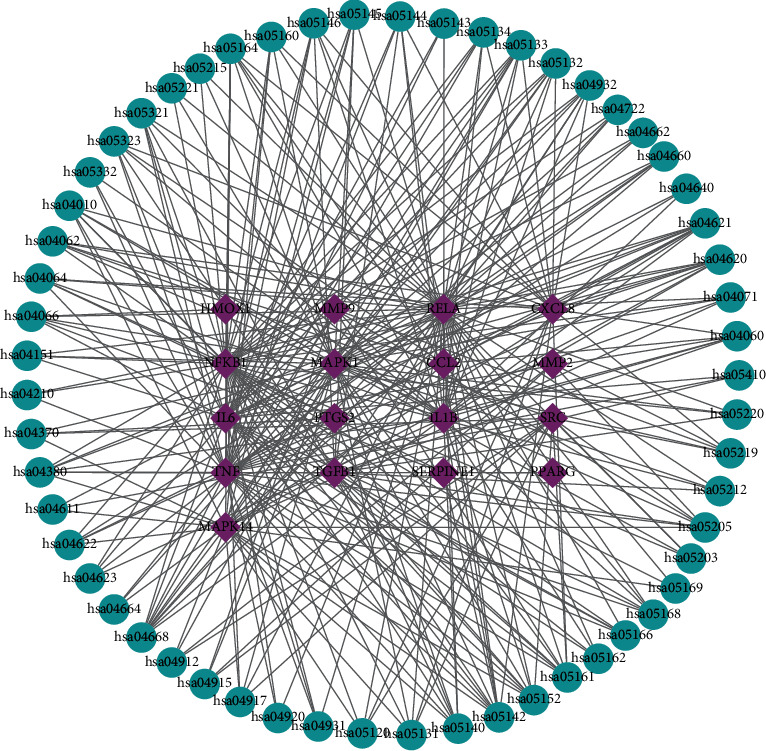
Target-pathway network. The blue ellipses display pathways. The purple diamonds display SMP targets related to GA.

To systematically and comprehensively disclose interactions of GA-related targets and pathways, target-pathway network was built by Cytoscape (3.6.0) software. The target-pathway network contained 74 nodes (17 hub targets and 57 pathways) and 311 edges (Figure 7). After network analysis, the topological parameters were calculated (Supplementary Table 9). Based on the degree values of pathways, 27 pathawys were identified as major pathways with degree more than the mean degree (5.46). Notably, the top 6 signaling pathways were Chagas disease (American trypanosomiasis) (hsa05142), TNF signaling pathway (hsa04668), NOD-like receptor signaling pathway (hsa04621), tuberculosis (hsa05152), hepatitis B (hsa05161), and influenza A (hsa05164).

Multiple studies focused that the approaches of treating GA lowered levels of uric acid and reduced inflammation [31]. Although Chagas disease (American trypanosomiasis), tuberculosis, hepatitis B, and influenza A were pathways associated with infection disease, the pathways had complex interactions. The four pathways related to infection disease had common subpathways, namely, the Toll-like receptor signaling pathway. The Toll-like receptor signaling pathway was implicated in MSU crystal-induced inflammatory cytokine release in monocytes/macrophages [32]. In addition, the JAK-STAT signaling pathway, NOD-like receptor signaling pathway, and MAPK signaling pathway in four pathways related to disease arose in high frequency. It was reported that MSU crystals could directly or indirectly activate the Toll-like receptor (TLRs) which recruited myeloid differentiation factor 88 (MyD88) in turn. Then, interleukin-1 receptor-related kinase (IRAK) and transformed growth factor-beta activated kinase (TAK1) were activated. Thereby, the IkB kinase cascade was triggered and NF- *κ*B was activated. Meanwhile, the MAPK signaling pathway was activated to increase the expression of proinflammatory molecules, for instance, tumor necrosis factor (TNF)-*α*, IL-6, and IL-8, resulting in inflammatory reaction. From the above analysis, SMP in treatment of GA might inhibit the occurrence of inflammatory reaction via the Toll-like receptor/NF-*κ*B/MAPK pathway [33]. In addition, recent studies had shown that the JAK-STAT signaling pathway [34], NOD-like receptor signaling pathway [35, 36], and TNF signaling pathway [37] were activated, causing secretion of proinflammatory and autoimmunity in the occurrence and development of GA. Pathogenesis of GA is associated with inflammation, immunity, and metabolism. Accordingly, SMP acted on anti-inflammation and immunity in treatment of GA via the Toll-like receptor signaling pathway, JAK-STAT signaling pathway, NOD-like receptor signaling pathway, MAPK signaling pathway, and so on.

These pathways associated with GA-relevant targets of SMP might become multiple inflammatory and immune-related pathways in the development of GA. Besides, we also found that some key targets acted on multiple pathways (e.g., NFKB1, RELA, MAPK1, and TNF acted on more than or equal to 35 pathways). NFKB1 and RELA, members of the NF-*κ*B family, were involved in the NF-*κ*B signaling pathway and TLR4-NF*κ*B-IL1b signaling pathway, respectively, which regulated the expressing pro-inflammatory cytokines and played a central role in the pathogenesis of acute inflammation in arthritis [38–40]. MAPK1 (also known as ERK2) and TNF were proinflammatory cytokines which contributed to inflammation of acute GA [41–43]. Taken together, NFKB1, RELA, MAPK1, and TNF might be considered as potential therapeutic targets in treating GA, but they also needed molecular docking for further verification.

### 3.9. Validation of Compound-Target Interaction

**Figure 8:**
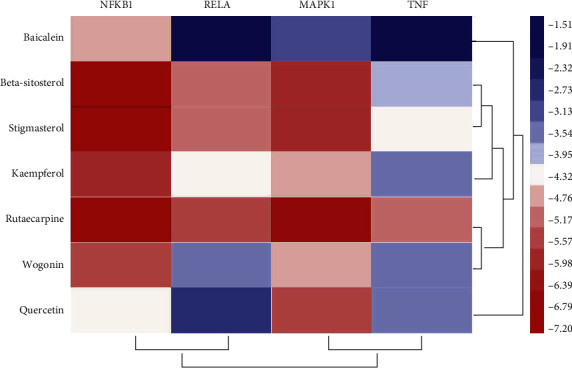
The heat map of molecular docking.

**Figure 9:**
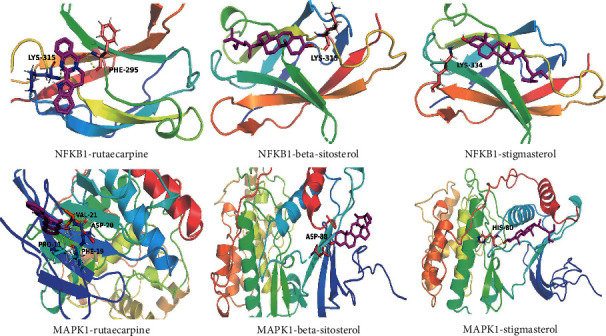
Molecular docking pattern.

The kernel compounds (quercetin, kaempferol, wogonin, rutaecarpine, baicalein, beta-sitosterol, and stigmasterol) and targets (NFKB1, RELA, MAPK1, and TNF) were obtained from the result of compound-target and target-pathway network analysis for the binding validation experiment. The binding energy was applied to assess the effects of compound-target interactions. The binding energy was lower, indicating that the molecular docking was more effective. In the molecular docking process, the compound-target interaction with docking energy less than 5 kcal/mol would deem the effective docking [44]. The result of molecular docking is shown in Supplementary Table 10. 11 compound-target interactions had binding efficiencies including rutaecarpine with NFKB1 (−7.20 kcal/mol), stigmasterol with NFKB1 (−6.91 kcal/mol), beta-sitosterol with NFKB1 (−6.84 kcal/mol), rutaecarpine with MAPK1 (−6.66 kcal/mol), beta-sitosterol with MAPK1(−5.87 kcal/mol), stigmasterol with MAPK1(−5.80 kcal/mol), kaempferol with NFKB1 (−5.74 kcal/mol), rutaecarpine with RELA (−5.43 kcal/mol), quercetin with MAPK1 (−5.43 kcal/mol), beta-sitosterol with RELA (−5.15 kcal/mol), and wogonin with NFKB1 (−5.40 kcal/mol). The heat map of molecular docking is portrayed in Figure 8. From the above results of molecular docking, we concluded that 5 compounds (beta-sitosterol, rutaecarpine, stigmasterol, kaempferol, and wogonin) possessed a better combination with NFKB1. Additionally, 4 compounds (rutaecarpine, stigmasterol,quercetin, and beta-sitosterol) had a close interaction with MAPK1, suggesting that NFKB1 and MAPK1 might be considered as therapeutic targets in treatment of GA. Partial molecular docking patterns are depicted in Figure 9.

## 4. Conclusions

In the present study, network pharmacology was proposed to dissect the molecular mechanism of SMP in treatment of GA. Our study found that HB potentially played a crucial role in the whole SMP formula. We also successfully identified that quercetin, kaempferol, wogonin, rutaecarpine, baicalein, beta-sitosterol, and stigmasterol could exert synergistic or antagonistic effects in treatment of GA through acting on multiple targets. Moreover, SMP took up significant position in anti-inflammatory by regulating multiple pathways. In addition, the results of molecular docking displayed the reliability and accuracy of the prediction results. In summary, the study had provided systematic and comprehensive sight into the mechanism of SMP in treating GA, which might contribute to the further application for SMP development.

## Figures and Tables

**Table 1:** 47 active compounds of SMP and their parameters.

Mol. ID	Chemical name	PubChem CID	OB (%)	DL	Herbs
MOL000085	Beta-daucosterol_qt	N/A	36.91	0.75	CZ, NX
MOL000088	Beta-sitosterol 3-O-glucoside_qt	N/A	36.91	0.75	CZ
MOL000092	Daucosterin_qt	N/A	36.91	0.76	CZ
MOL000094	Daucosterol_qt	N/A	36.91	0.76	CZ
MOL000098	Quercetin	5280343	46.43	0.28	HB, NX
MOL000173	Wogonin	5281703	30.68	0.23	CZ, NX
MOL000184	NSC63551	5356634	39.25	0.76	CZ
MOL000186	Stigmasterol 3-O-beta-D-glucopyranoside_qt	N/A	43.83	0.76	CZ
MOL000188	3*β*-Acetoxyatractylone	N/A	40.57	0.22	CZ
MOL000358	Beta-sitosterol	222284	36.91	0.75	HB, NX
MOL000422	Kaempferol	5280863	41.88	0.24	NX
MOL000449	Stigmasterol	5280794	43.83	0.76	HB, NX
MOL000622	Magnograndiolide	5319198	63.71	0.19	HB
MOL000762	Palmidin A	N/A	35.36	0.65	HB
MOL000785	Palmatine	19009	64.6	0.65	HB, NX
MOL000787	Fumarine	N/A	59.26	0.83	HB
MOL000790	Isocorypalmine	440229	35.77	0.59	HB
MOL001006	Poriferasta-7,22E-dien-3beta-ol	5283663	42.98	0.76	NX
MOL001131	Phellamurin_qt	N/A	56.6	0.39	HB
MOL001454	Berberine	N/A	36.86	0.78	HB, NX
MOL001455	(S)-canadine	N/A	53.83	0.77	HB
MOL001458	Coptisine	N/A	30.67	0.86	HB, NX
MOL001771	Poriferast-5-en-3beta-ol	457801	36.91	0.75	HB
MOL002641	Phellavin_qt	N/A	35.86	0.44	HB
MOL002643	Delta-7-stigmastenol	12315385	37.42	0.75	HB, NX
MOL002644	Phellopterin	98608	40.19	0.28	HB
MOL002651	Dehydrotanshinone II A	128994	43.76	0.4	HB
MOL002660	Niloticin	44559946	41.41	0.82	HB
MOL002662	Rutaecarpine	65752	40.3	0.6	HB
MOL002663	Skimmianin	6760	40.14	0.2	HB
MOL002666	Chelerythrine	N/A	34.18	0.78	HB
MOL002668	Worenine	20055073	45.83	0.87	HB
MOL002670	Cavidine	193148	35.64	0.81	HB
MOL002671	Candletoxin A	442008	31.81	0.69	HB
MOL002672	Hericenone H	N/A	39	0.63	HB
MOL002714	Baicalein	5281605	33.52	0.21	NX
MOL002776	Baicalin	64982	40.12	0.75	NX
MOL002894	Berberrubine	72703	35.74	0.73	HB
MOL002897	Epiberberine	N/A	43.09	0.78	NX
MOL003847	Inophyllum E	N/A	38.81	0.85	NX
MOL004355	Spinasterol	5281331	42.98	0.76	NX
MOL005438	Campesterol	173183	37.58	0.71	HB
MOL006413	Phellochin	189290	35.41	0.82	HB
MOL006422	Thalifendine	3084288	44.41	0.73	HB
MOL012461	28-Norolean-17-en-3-ol	N/A	35.93	0.78	NX
MOL012537	Spinoside A	5281325	41.75	0.4	NX
MOL012542	*β*-Ecdysterone	27545171	44.23	0.82	NX

## Data Availability

The original data used to support the findings of this study are included within the supplementary information file.

## Supplementary Materials

Supplementary Materials Supplementary Table 1: compounds in SMP. Supplementary Table 2: target-related active compounds. Supplementary Table 3: the topological parameters of the herb-compound-target network. Supplementary Table 4: GA-related targets. Supplementary Table 5: the topological parameters of the compound-target network. Supplementary Table 6: the topological parameters of the protein-protein network. Supplementary Table 7: the results of GOBP enrichment analysis. Supplementary Table 8: the results of KEGG pathway enrichment analysis. Supplementary Table 9: the topological parameters of the target-pathway network. Supplementary Table S10: the result of molecular docking.
