# Supplementary material for: Study on the Multitarget Mechanism of Sanmiao Pill on Gouty Arthritis Based on Network Pharmacology
Source: Evid Based Complement Alternat Med. 2020 Aug 4;2020:9873739. doi: 10.1155/2020/9873739 (PMC7424379; doi:10.1155/2020/9873739)
Supplement: Supplementary Materials — Supplementary Table 1: compounds in SMP. Supplementary Table 2: target-related active compounds. Supplementary Table 3: the topological parameters of the herb-compound-target network. Supplementary Table 4: GA-related targets. Supplementary Table 5: the topological parameters of the compound-target network. Supplementary Table 6: the topological parameters of the protein-protein network. Supplementary Table 7: the results of GOBP enrichment analysis. Supplementary Table 8: the results of KEGG pathway enrichment analysis. Supplementary Table 9: the topological parameters of the target-pathway network. Supplementary Table S10: the result of molecular docking. [file 9873739.f1.docx]

**Supplementary Table 1 Compounds in SMP**

| **Chemical Name** | **Herbs** |
| --- | --- |
| alpha-humulene | CZ |
| beta-Eudesmol | CZ |
| 2-[(1R,3S,4S)-3-isopropenyl-4-methyl-4-vinylcyclohexyl]propan-2-ol | CZ |
| atractylenolide i | CZ |
| atractylenolideII | CZ |
| selina-4(14),7(11)-dien-8-one | CZ |
| (24S)-5beta-Stigmastan-3beta-ol | CZ |
| beta-sitosterol 3-O-glucoside | CZ |
| beta-sitosterol 3-O-glucoside_qt | CZ |
| daucosterin | CZ |
| daucosterin_qt | CZ |
| daucosterol | CZ |
| daucosterol_qt | CZ |
| delta 7-stigmastenol | CZ |
| vanillic acid | CZ |
| beta-Chamigrene | CZ |
| Atractylodin | CZ |
| atractylone | CZ |
| 2-[(2S,5S,6S)-6,10-dimethylspiro[4.5]dec-9-en-2-yl]propan-2-ol | CZ |
| ZINC01609418 | CZ |
| 3β-hydroxyatractylone | CZ |
| ()-2-Carene | CZ |
| alpha-Guaiene | CZ |
| guaiene | CZ |
| Guaiol | CZ |
| (2E,8E)-9-(2-furyl)nona-2,8-dien-4,6-diyn-1-ol | CZ |
| cyperene | CZ |
| atractylenolide iii | CZ |
| 2-Hydroxyisoxypropyl-3-hydroxy-7-isopentene-2,3-dihydrobenzofuran-5-carboxylic | CZ |
| Aractylenolide II | CZ |
| atractylenolide III | CZ |
| Atractyloyne | CZ |
| Beta- Eudesmol | CZ |
| NSC63551 | CZ |
| Stigmasterol 3-O-beta-D-glucopyranoside | CZ |
| Stigmasterol 3-O-beta-D-glucopyranoside_qt | CZ |
| butenolide B | CZ |
| 3β-acetoxyatractylone | CZ |
| acetyl atractylodinol | CZ |
| 3,5-dimethoxy-4-glucosyloxyphenylallylalcohol | CZ |
| 3,5-dimethoxy-4-glucosyloxyphenylallylalcohol_qt | CZ |
| 2-(1,4a-dimethyl-2,3-dihydroxydecahydronaphthalen-7-yl)isopropyl glucoside | CZ |
| (Z)-caryophyllene | CZ |
| patchoulene | CZ |
| Oroxindin | CZ |
| Furol | CZ, NX |
| beta-daucosterol | CZ, NX |
| beta-daucosterol_qt | CZ, NX |
| wogonin | CZ, NX |
| palmitic acid | HB |
| Undecenal | HB |
| Myrcene | HB |
| (S)-(+)-alpha-Phellandrene | HB |
| L-Limonen | HB |
| eugenol | HB |
| Syrigin | HB |
| 4-[(Z)-3-hydroxyprop-1-enyl]-2,6-dimethoxyphenol | HB |
| FERULIC ACID (CIS) | HB |
| Friedelin | HB |
| Magnograndiolide | HB |
| vanillin | HB |
| PENTYLFURAN | HB |
| oleic acid | HB |
| trans-2-nonenal | HB |
| (2S,3S)-3,5,7-trihydroxy-2-(4-hydroxyphenyl)chroman-4-one | HB |
| Palmidin A | HB |
| magnoflorine | HB |
| Menisporphine | HB |
| STOCK1N-14407 | HB |
| Fumarine | HB |
| jatrorrizine | HB |
| Isocorypalmine | HB |
| menisperine | HB |
| paeonol | HB |
| beta-elemene | HB |
| Mnk | HB |
| phellamurin_qt | HB |
| Pisol | HB |
| Oxophorone | HB |
| PENTADECYLIC ACID | HB |
| (S)-Canadine | HB |
| columbamine | HB |
| EUG | HB |
| poriferast-5-en-3beta-ol | HB |
| Isovanillin | HB |
| Dauricine (8CI) | HB |
| Methyl 3-furoate | HB |
| Javanicin | HB |
| N-Methylflindersine | HB |
| Homocresol | HB |
| (s)-carvone | HB |
| beta-Rhodinol | HB |
| Phlorol | HB |
| (±)-lyoniresinol | HB |
| Kihadalactone A | HB |
| Obacunoic acid | HB |
| Obamegine | HB |
| phellavin | HB |
| Phellavin_qt | HB |
| phellodendrine | HB |
| Phellopterin | HB |
| PEA | HB |
| Vanilloloside | HB |
| Vanillyl alcohol | HB |
| (4R)-limonene 1beta,2beta-epoxide | HB |
| Coniferin | HB |
| Coniferol | HB |
| Dehydrotanshinone II A | HB |
| delta7-Dehydrosophoramine | HB |
| Dictamine | HB |
| Amurensin | HB |
| Amurensin_qt | HB |
| dihydroniloticin | HB |
| hispidol B | HB |
| kihadalactone B | HB |
| kihadanin A | HB |
| niloticin | HB |
| nomilin | HB |
| rutaecarpine | HB |
| Skimmianin | HB |
| Fagarine | HB |
| Ferulic Acid | HB |
| Chelerythrine | HB |
| Worenine | HB |
| Campesteryl ferulate | HB |
| Cavidine | HB |
| Candletoxin A | HB |
| Hericenone H | HB |
| Hispidone | HB |
| magnoflorine | HB |
| berberrubine | HB |
| Noroxyhydrastinine | HB |
| phellodendrine | HB |
| Ethyl caffeate | HB |
| Guasol | HB |
| IPH | HB |
| nonanoic acid | HB |
| dodec-2-enal | HB |
| naphthalene | HB |
| limonin | HB |
| 2-PENTADECANONE | HB |
| 5-METHYLFURFURAL | HB |
| Maruzen M | HB |
| o-cresol | HB |
| CREOSOL | HB |
| Methyl naphthalene | HB |
| campesterol | HB |
| isoferulic acid | HB |
| Cyclopentenone | HB |
| Methyl caffeate | HB |
| Clorius | HB |
| Ptelein | HB |
| SMR000232320 | HB |
| Canthin-6-one | HB |
| 4,10-dimethylene-7-isopropyl-5(E)-cyclodecenol | HB |
| 4-[(1R,3aS,4R,6aS)-4-(4-hydroxy-3,5-dimethoxyphenyl)-1,3,3a,4,6,6a-hexahydrofuro[4,3-c]furan-1-yl]-2,6-dimethoxyphenol | HB |
| guanidine | HB |
| melianone | HB |
| phellochin | HB |
| 7-hydroxy-6-(2-hydroxyethyl)coumarin | HB |
| thalifendine | HB |
| vanilloloside | HB |
| Furfuranol | HB |
| cis-9-Hexadecenal | HB |
| (S)-4-Nonanolide | HB |
| 2,4,6-trimethyl-Octane | HB |
| Methyl atratate | HB |
| candicine | HB |
| 2-undecenoic acid | HB |
| Homoveratrole | HB |
| Obacunone | HB |
| Auraptene | HB |
| quercetin | HB, NX |
| caprylic acid | HB, NX |
| Sitogluside | HB, NX |
| beta-sitosterol | HB, NX |
| D-Galacturonic acid, homopolymer | HB, NX |
| Stigmasterol | HB, NX |
| WLN: VHR | HB, NX |
| palmatine | HB, NX |
| stearic acid | HB, NX |
| methyl palmitate | HB, NX |
| berberine | HB, NX |
| coptisine | HB, NX |
| delta 7-stigmastenol | HB, NX |
| Hyperin | HB, NX |
| Acetylfuran | HB, NX |
| DIBP | NX |
| palmitic acid | NX |
| oleanolic acid | NX |
| succinic acid | NX |
| rutin | NX |
| kaempferol | NX |
| betaine | NX |
| Astragalin | NX |
| hexanal | NX |
| 1-hexanol | NX |
| DBP | NX |
| 2-heptanone | NX |
| (E)-oct-3-en-2-one | NX |
| HMF | NX |
| EEE | NX |
| OXA | NX |
| Dekan | NX |
| hexadecane | NX |
| Heptadekan | NX |
| Henicosane | NX |
| (6R,10R)-6,10,14-trimethylpentadecan-2-one | NX |
| Tricosane | NX |
| Dodekan | NX |
| tetradecane | NX |
| poriferasta-7,22E-dien-3beta-ol | NX |
| octanol | NX |
| Azelex | NX |
| myristic acid | NX |
| Oktadekan | NX |
| TWT | NX |
| UPL | NX |
| oleanolic acid-3-O-β-D-glucuronopyranoside_qt | NX |
| Crysophanol | NX |
| Tetracosane | NX |
| n-butyl-β-D-fructopyronoside | NX |
| hexanoic acid | NX |
| Chrysophanol-8-O-beta-D-(6'-O-galloyl)-glucopyranoside | NX |
| (R)-Allantoin | NX |
| PTL | NX |
| baicalein | NX |
| Baicalin | NX |
| epiberberine | NX |
| Amylol | NX |
| pent-3-en-2-one | NX |
| Heptanol | NX |
| geniposide_qt | NX |
| pjs-1_qt | NX |
| Inophyllum E | NX |
| Spinasterol | NX |
| (2R,3R,4S,5S,6R)-2-[[(3S,5S,9R,10S,13R,14R,17R)-17-[(E,2R,5S)-5-ethyl-6-methylhept-3-en-2-yl]-10,13-dimethyl-2,3,4,5,6,9,11,12,14,15,16,17-dodecahydro-1H-cyclopenta[a]phenanthren-3-yl]oxy]-6-(hydroxymethyl)oxane-3,4,5-triol | NX |
| heptanoic acid | NX |
| Nonenone | NX |
| ginsenoside Ro_qt | NX |
| ginsenoside Ro | NX |
| Areginal | NX |
| (2S,3S,4S,5R,6R)-6-[[(3S,4aR,6aR,6bS,8aS,12aS,14aR,14bR)-4,4,6a,6b,11,11,14b-heptamethyl-8a-[oxo-[[(2S,3R,4S,5S,6R)-3,4,5-trihydroxy-6-(hydroxymethyl)-2-tetrahydropyranyl]oxy]methyl]-1,2,3,4a,5,6,7,8,9,10,12,12a,14,14a-tetradecahydropicen-3-yl]oxy]-3,4-di | NX |
| 2,6-Dimethylpiazine | NX |
| Amyl ketone | NX |
| PRZ | NX |
| acetaldehyde | NX |
| chikusetsusaponin Ⅳa | NX |
| 3-epioleanolic acid | NX |
| ginsenoside-Rg2_qt | NX |
| IPZ | NX |
| 3-O-(β-D-glucopyranosiduronic acid) oleanolic acid | NX |
| (20r,22r)-2beta,3beta,20,22,26-pentahydroxy-cholestan-7,12-dien-6-one | NX |
| (20R,22R)-2β,3β,20,22,26-pentahydroxy-cholestan-7,12-dien-6-one | NX |
| 1. (β-D-Oxyglucose)-28-Oxo-12-oleanolic acid-3β-3-O-(β-D-glucose)-β-D 2. -glucuronic acid methylester | NX |
| 18-(β-D-Oxyglucose)-28-Oxo-12-oleanolic acid-3β-3-O-(β-D-glucose)-β-D-glucuronic acid methylester_qt | NX |
| oct-1-en-2-ol | NX |
| Inokosterone | NX |
| 28-deglucosyl achyranthoside D methyl ester | NX |
| achyranthoside D trimethyl ester_qt | NX |
| 28-deglucosyl-chikusetsusaponin,iva | NX |
| 28-deglucosyl-chikusetsusaponin,iva_qt | NX |
| 28-norolean-17-en-3-ol | NX |
| 2-Octenal, 2-butyl- | NX |
| 2β,3β,20α,22α,25-pentahydroxy cholesta-8,14-dien-6-one | NX |
| 3-O-(β-D-glucose)-oleanolic acid-28-O-(β-D-glucose) | NX |
| (3S,4aR,6aR,6bS,8aS,12aS,14aR,14bR)-4,4,6a,6b,11,11,14b-heptamethyl-1,2,3,4a,5,6,7,8,9,10,12,12a,14,14a-tetradecahydropicene-3,8a-diol | NX |
| 3-O-(β-D-glucuronic acid)-oleanolic acid-28-O-(β-D-glucose) | NX |
| 3-O-β-D-glucopyranosyl-α-spinalsterol | NX |
| 3-O-β-D-glucuronopyranoside-6-O-butyl ester | NX |
| 3-O-β-D-glucuronopyranoside-6-O-methyl ester | NX |
| 6-Dodecanone | NX |
| achybidensaponin,i | NX |
| achybidensaponin,i_qt | NX |
| achybidensaponin,ii | NX |
| achybidensaponin,ii_qt | NX |
| achyranthesterone A | NX |
| achyranthoside Ⅱ | NX |
| achyranthoside Ⅱ_qt | NX |
| achyranthoside Ⅲ | NX |
| achyranthoside Ⅳ | NX |
| achyranthoside A | NX |
| achyranthoside A_qt | NX |
| achyranthoside A trimethyl ester | NX |
| achyranthoside A trimethyl ester_qt | NX |
| achyranthoside c | NX |
| achyranthoside c_qt | NX |
| achyranthoside C butyl dimethyl ester | NX |
| achyranthoside C butyl dimethyl ester_qt | NX |
| achyranthoside C dimethyl ester | NX |
| achyranthoside C dimethyl ester_qt | NX |
| achyranthoside C trimethyl ester | NX |
| achyranthoside D | NX |
| achyranthoside D trimethyl ester | NX |
| achyranthoside E | NX |
| achyranthoside E_qt | NX |
| achyranthoside E butyl dimethyl ester | NX |
| achyranthoside E butyl dimethyl ester_qt | NX |
| achyranthoside E dimethyl ester | NX |
| achyranthoside E dimethyl ester_qt | NX |
| achyranthoside E trimethyl ester | NX |
| achyranthoside E trimethyl ester_qt | NX |
| achyranthoside I | NX |
| bidentatoside,i | NX |
| bidentatoside,i_qt | NX |
| bidentatoside,ii | NX |
| bidentatoside,ii_qt | NX |
| chikusetsusaponin Ⅳ | NX |
| chikusetsusaponin I | NX |
| chikusetsusaponin IVA butyl ester | NX |
| Chikusetsusaponin IVa methyl ester | NX |
| Chikusetsusaponin V butyl ester | NX |
| Chikusetsusaponin V methyl ester | NX |
| deglucose chikusetsusaponin Iva | NX |
| deglucose chikusetsusaponin Iva_qt | NX |
| Ecdysterone-3-O-beta-D-glucopyranoside | NX |
| Ecdysterone-3-O-beta-D-glucopyranoside_qt | NX |
| geniposide | NX |
| hederagenin-28-O-β-D-glucopyranosyl ester | NX |
| hederagenin-28-O-β-D-glucopyranosyl ester_qt | NX |
| Monoglucuronide F | NX |
| niuxixinsterone A | NX |
| niuxixinsterone B | NX |
| niuxixinsterone C | NX |
| N-trans-feruloyl-3-methoxytyramine-4'-O-β-D-glucopyranoside | NX |
| Hmp-hmpep | NX |
| N-trans-feruloyl-3-methoxytyramine-4-O-β-D-glucopyranoside | NX |
| oleanolic acid 3-O-[β-D-glucuronopyranoside-6-O-butyl ester]  -28-O-β-D-glucopyranoside | NX |
| oleanolic acid 3-O-[β-D-glucuronopyranoside-6-O-ethyl ester]  -28-O-β-D-glucopyranoside | NX |
| oleanolic acid 3-O-[β-D-glucuronopyranoside-6-O-methyl ester]  -28-O-β-D-glucopyranoside | NX |
| oleanolic acid-3-O-β-D-(6'-butyl)-glucuronopyranoside | NX |
| oleanolic acid-3-O-β-D-glucuronopyranoside | NX |
| pjs-1 | NX |
| Polypodine B | NX |
| quercetin-3-O-rutinoside | NX |
| rhapontisterone B | NX |
| rubrosterone | NX |
| Rubschisantherin | NX |
| Spinoside A | NX |
| stachysterone A | NX |
| stachysterone D | NX |
| zingibroside r1 | NX |
| zingibroside r1_qt | NX |
| β-ecdysterone | NX |
| Furol | HB |

**Supplementary Table2 Targets related active compounds**

| **Target** | **Common name** | **Uniprot ID** |
| --- | --- | --- |
| P-glycoprotein 1 | ABCB1 | P08183 |
| Multidrug resistance-associated protein 1 | ABCC1 | P33527 |
| ATP-binding cassette sub-family G member 2 | ABCG2 | Q9UNQ0 |
| Acetyl-CoA carboxylase 1 | ACACA | Q13085 |
| Acetylcholinesterase | ACHE | P22303 |
| Prostatic acid phosphatase | ACPP | P15309 |
| Alcohol dehydrogenase 1C | ADH1C | P00326 |
| Adenosine A1 receptor | ADORA1 | P30542 |
| Adenosine A2a receptor | ADORA2A | P29274 |
| Alpha-1A adrenergic receptor | ADRA1A | P35348 |
| Alpha-1B adrenergic receptor | ADRA1B | P35368 |
| Alpha-1D adrenergic receptor | ADRA1D | P25100 |
| Alpha-2A adrenergic receptor | ADRA2A | P08913 |
| Alpha-2B adrenergic receptor | ADRA2B | P18089 |
| Alpha-2C adrenergic receptor | ADRA2C | P18825 |
| Beta-1 adrenergic receptor | ADRB1 | P08588 |
| Beta-2 adrenergic receptor | ADRB2 | P07550 |
| Aryl hydrocarbon receptor | AHR | P35869 |
| Activator of 90 kDa heat shock protein ATPase homolog 1 | AHSA1 | O95433 |
| Aldehyde reductase | AKR1A1 | P14550 |
| Aldose reductase | AKR1B1 | P15121 |
| Aldo-keto reductase family 1 member C1 | AKR1C1 | Q04828 |
| Aldo-keto reductase family 1 member C2 | AKR1C2 | P52895 |
| Aldo-keto reductase family 1 member C3 | AKR1C3 | P42330 |
| Aldo-keto reductase family 1 member C4 | AKR1C4 | P17516 |
| RAC-alpha serine/threonine-protein kinase | AKT1 | P31749 |
| ALK tyrosine kinase receptor | ALK | Q9UM73 |
| Arachidonate 12-lipoxygenase | ALOX12 | P18054 |
| Arachidonate 15-lipoxygenase | ALOX15 | P16050 |
| Arachidonate 5-lipoxygenase | ALOX5 | P09917 |
| Annexin A1 | ANXA1 | P04083 |
| DNA-(apurinic or apyrimidinic site) lyase | APEX1 | P27695 |
| Apolipoprotein D | APOD | P05090 |
| Androgen receptor | AR | P10275 |
| Arginase-1 | ARG1 | P05089 |
| Serine/threonine-protein kinase Aurora-B | AURKB | Q96GD4 |
| Vasopressin V2 receptor | AVPR2 | P30518 |
| Tyrosine-protein kinase receptor UFO | AXL | P30530 |
| Beta-secretase 1 | BACE1 | P56817 |
| Bcl-2-binding component 3 | BAD | Q92934 |
| Apoptosis regulator BAX | BAX | Q07812 |
| Cholinesterase | BCHE | P06276 |
| Apoptosis regulator Bcl-2 | BCL2 | P10415 |
| Bcl-2-like protein 1 | BCL2L1 | Q07817 |
| Baculoviral IAP repeat-containing protein 5 | BIRC5 | O15392 |
| Carbonic anhydrase I | CA1 | P00915 |
| Carbonic anhydrase XII | CA12 | O43570 |
| Carbonic anhydrase XIII | CA13 | Q8N1Q1 |
| Carbonic anhydrase XIV | CA14 | Q9ULX7 |
| Carbonic anhydrase II | CA2 | P00918 |
| Carbonic anhydrase III | CA3 | P07451 |
| Carbonic anhydrase IV | CA4 | P22748 |
| Carbonic anhydrase VA | CA5A | P35218 |
| Carbonic anhydrase VI | CA6 | P23280 |
| Carbonic anhydrase VII | CA7 | P43166 |
| Carbonic anhydrase IX | CA9 | Q16790 |
| Voltage-dependent L-type calcium channel subunit alpha-1S | CACNA1S | Q13698 |
| Calmodulin | CALM1 | P0DP23 |
| CaM kinase II beta | CAMK2B | Q13554 |
| Caspase-3 | CASP3 | P42574 |
| Caspase-8 | CASP8 | Q14790 |
| Caspase-9 | CASP9 | P55211 |
| Caveolin-1 | CAV1 | Q03135 |
| Carbonyl reductase [NADPH] 1 | CBR1 | P16152 |
| C-C motif chemokine 2 | CCL2 | P13500 |
| G2/mitotic-specific cyclin-B1 | CCNB1 | P14635 |
| G1/S-specific cyclin-D1 | CCND1 | P24385 |
| CD40 ligand | CD40LG | P29965 |
| Cyclin-dependent kinase 1 | CDK1 | P06493 |
| Cell division protein kinase 2 | CDK2 | P24941 |
| Cyclin-dependent kinase 5 activator 1 | CDK5R1 | Q15078 |
| Cyclin-dependent kinase 6 | CDK6 | Q00534 |
| Cyclin-dependent kinase inhibitor 1 | CDKN1A | P38936 |
| Cyclin-dependent kinase inhibitor 2A, isoforms 1/2/3 | CDKN2A | P42771 |
| Serine/threonine-protein kinase Chk1 | CHEK1 | O14757 |
| Serine/threonine-protein kinase Chk2 | CHEK2 | O96017 |
| Cholesterol oxidase | choB | P22637 |
| Muscarinic acetylcholine receptor M1 | CHRM1 | P11229 |
| Muscarinic acetylcholine receptor M2 | CHRM2 | P08172 |
| Muscarinic acetylcholine receptor M3 | CHRM3 | P20309 |
| Muscarinic acetylcholine receptor M4 | CHRM4 | P08173 |
| Muscarinic acetylcholine receptor M5 | CHRM5 | P08912 |
| Neuronal acetylcholine receptor subunit alpha-2 | CHRNA2 | Q15822 |
| Neuronal acetylcholine receptor subunit alpha-7 | CHRNA7 | P36544 |
| Inhibitor of nuclear factor kappa-B kinase subunit alpha | CHUK | O15111 |
| Claudin-4 | CLDN4 | O14493 |
| C-type lectin domain family 4 member E | CLEC4E | Q9ULY5 |
| Collagen alpha-1(I) chain | COL1A1 | P02452 |
| Collagen alpha-1(III) chain | COL3A1 | P02461 |
| Cell division control protein 2 homolog | CRK2 | Q07785 |
| C-reactive protein | CRP | P02741 |
| Casein kinase II alpha | CSNK2A1 | P68400 |
| Chymotrypsinogen B | CTRB1 | P17538 |
| Cathepsin D | CTSD | P07339 |
| C-X-C motif chemokine 10 | CXCL10 | P02778 |
| C-X-C motif chemokine 11 | CXCL11 | O14625 |
| C-X-C motif chemokine 2 | CXCL2 | P19875 |
| Interleukin-8 | CXCL8 | P10145 |
| Interleukin-8 receptor A | CXCR1 | P25024 |
| CytochromeC | CYCS | P99999 |
| Steroid 17-alpha-hydroxylase/17,20 lyase | CYP17A1 | P05093 |
| Cytochrome P450 19A1 | CYP19A1 | P11511 |
| Cytochrome P450 1A1 | CYP1A1 | P04798 |
| Cytochrome P450 1A2 | CYP1A2 | P05177 |
| Cytochrome P450 1B1 | CYP1B1 | Q16678 |
| Cytochrome P450 2B6 | CYP2B6 | P20813 |
| Cytochrome P450 2D6 | CYP2D6 | P10635 |
| Cytochrome P450 3A4 | CYP3A4 | P08684 |
| Cytochrome P450 3A4 | CYP3A5 | P08685 |
| Cytochrome P450 51 | CYP51A1 | Q16850 |
| Death-associated protein kinase 1 | DAPK1 | P53355 |
| DDB1- and CUL4-associated factor 5 | DCAF1 | Q9Y4B6 |
| Type I iodothyronine deiodinase | DIO1 | P49895 |
| DNA | DNA | Nucleotide |
| Dipeptidyl peptidase IV | DPP4 | P27487 |
| Dopamine D1 receptor | DRD1 | P21728 |
| Dopamine D2 receptor | DRD2 | P14416 |
| D(3) dopamine receptor | DRD3 | P35462 |
| Dopamine D4 receptor | DRD4 | P21917 |
| D(1B) dopamine receptor | DRD5 | P21918 |
| Dual oxidase 2 | DUOX2 | Q9NRD8 |
| Transcription factor E2F1 | E2F1 | Q01094 |
| Transcription factor E2F2 | E2F2 | Q14209 |
| Pro-epidermal growth factor | EGF | P01133 |
| Epidermal growth factor receptor | EGFR | P00533 |
| Egl nine homolog 1 | EGLN1 | Q9GZT9 |
| Eukaryotic translation initiation factor 6 | EIF6 | P56537 |
| ETS domain-containing protein Elk-1 | ELK1 | P19419 |
| Receptor tyrosine-protein kinase erbB-2 | ERBB2 | P04626 |
| Receptor tyrosine-protein kinase erbB-3 | ERBB3 | P21860 |
| Estrogen receptor alpha | ESR1 | P03372 |
| Estrogen receptor beta | ESR2 | Q92731 |
| Steroid hormone receptor ERR1 | ESRRA | P11474 |
| Steroid hormone receptor ERR2 | ESRRB | O95718 |
| Coagulation factor Xa | F10 | P00742 |
| Thrombin | F2 | P00734 |
| Coagulation factor VII/tissue factor | F3 | P13726 |
| Coagulation factor VII | F7 | P08709 |
| Fatty acid-binding protein, epidermal | FABP5 | Q01469 |
| Tyrosine-protein kinase receptor FLT3 | FLT3 | P36888 |
| Fibronectin | FN1 | P02751 |
| Proto-oncogene c-Fos | FOS | P01100 |
| Fos-related antigen 1 | FOSL1 | P15407 |
| Fos-related antigen 2 | FOSL2 | P15408 |
| GABA-A receptor (anion channel) (Protein Group) | GABR(Protein Group) | N/A |
| Gamma-aminobutyric acid receptor subunit alpha-1 | GABRA1 | P14867 |
| Gamma-aminobutyric-acid receptor alpha-2 subunit | GABRA2 | P47869 |
| Gamma-aminobutyric-acid receptor subunit alpha-6 | GABRA6 | Q16445 |
| Gamma-aminobutyric-acid receptor alpha-3 subunit | GABRB3 | P47869 |
| Gap junction alpha-1 protein | GJA1 | P17302 |
| Glyoxalase I | GLO1 | Q04760 |
| G-protein coupled receptor 35 | GPR35 | Q9HC97 |
| Glutamate receptor 2 | GRIA2 | P42262 |
| NMDA receptor (Protein Group) | GRIN(Protein Group) | N/A |
| G protein-coupled receptor kinase 6 | GRK6 | P43250 |
| Glycogen synthase kinase-3 beta | GSK3B | P49841 |
| Glutathione S-transferase Mu 1 | GSTM1 | P09488 |
| Glutathione S-transferase Mu 2 | GSTM2 | P28161 |
| Glutathione S-transferase P | GSTP1 | P09211 |
| DNA gyrase subunit B | gyrB | P0AES6 |
| Hyaluronan synthase 2 | HAS2 | Q92819 |
| Histone deacetylase 2 | HDAC2 | Q92769 |
| Probable E3 ubiquitin-protein ligase HERC5 | HERC5 | Q9UII4 |
| Hypoxia-inducible factor 1-alpha | HIF1AN | Q9NWT6 |
| Hexokinase-2 | HK2 | P52789 |
| HMG-CoA reductase | HMGCR | P04035 |
| Heme oxygenase 1 | HMOX1 | P09601 |
| Corticosteroid 11-beta-dehydrogenase isozyme 1 | HSD11B1 | P28845 |
| Corticosteroid 11-beta-dehydrogenase isozyme 2 | HSD11B2 | P80365 |
| Estradiol 17-beta-dehydrogenase 1 | HSD17B1 | P14061 |
| Estradiol 17-beta-dehydrogenase 2 | HSD17B2 | P37059 |
| 3 beta-hydroxysteroid dehydrogenase/Delta 5-->4-isomerase type 1 | HSD3B1 | P14060 |
| Heat shock factor protein 1 | HSF1 | Q00613 |
| 78 kDa glucose-regulated protein | HSPA5 | P11021 |
| Heat shock protein beta-1 | HSPB1 | P04792 |
| Serotonin 1a (5-HT1a) receptor | HTR1A | P08908 |
| 5-hydroxytryptamine 2A receptor | HTR2A | P28223 |
| Serotonin 2b (5-HT2b) receptor | HTR2B | P41595 |
| 5-hydroxytryptamine 2C receptor | HTR2C | P28335 |
| 5-hydroxytryptamine receptor 3A | HTR3A | P46098 |
| Serotonin 7 (5-HT7) receptor | HTR7 | P34969 |
| Intercellular adhesion molecule 1 | ICAM1 | P05362 |
| Interferon gamma | IFNG | P01579 |
| Insulin-like growth factor I receptor | IGF1R | P08069 |
| Insulin-like growth factor II | IGF2 | P01344 |
| Insulin-like growth factor-binding protein 3 | IGFBP3 | P17936 |
| Ig gamma-1 chain C region | IGHG1 | P01857 |
| Inhibitor of nuclear factor kappa-B kinase subunit beta | IKBKB | O14920 |
| Interleukin-10 | IL10 | P22301 |
| Interleukin-1 alpha | IL1A | P01583 |
| Interleukin-1 beta | IL1B | P01584 |
| Interleukin-2 | IL2 | P60568 |
| Interleukin-4 | IL4 | P05112 |
| Interleukin-6 | IL6 | P05231 |
| Insulin receptor | INSR | P06213 |
| Interferon regulatory factor 1 | IRF1 | P10914 |
| Transcription factor AP-1 | JUN | P05412 |
| Potassium voltage-gated channel subfamily H member 2 | KCNH2 | Q12809 |
| Calcium-activated potassium channel subunit alpha 1 | KCNMA1 | Q12791 |
| Lysine-specific demethylase 4D-like | KDM4E | B2RXH2 |
| Vascular endothelial growth factor receptor 2 | KDR | P35968 |
| Keratin, type I cytoskeletal 12 | KRT12 | Q99456 |
| Keratin, type II cytoskeletal 2 epidermal | KRT2 | P35908 |
| Steroid Delta-isomerase | ksi | P07445 |
| Leukotriene A-4 hydrolase | LKHA4 | P09960 |
| Lipoprotein lipase | LPL | P06858 |
| Monoamine oxidase A | MAOA | P21397 |
| Amine oxidase [flavin-containing] B | MAOB | P27338 |
| Microtubule-associated protein 2 | MAP2 | P11137 |
| Mitogen-activated protein kinase 1 | MAPK1 | P28482 |
| Mitogen-activated protein kinase 14 | MAPK14 | Q16539 |
| Mitogen-activated protein kinase 8 | MAPK8 | P45983 |
| C-Jun-amino-terminal kinase-interacting protein 1 | MAPK8IP1 | Q9UQF2 |
| Microtubule-associated protein tau | MAPT | P10636 |
| Induced myeloid leukemia cell differentiation protein Mcl-1 | MCL1 | Q07820 |
| Hepatocyte growth factor receptor | MET | P08581 |
| Maltase-glucoamylase, intestinal | MGAM | O43451 |
| Interstitial collagenase | MMP1 | P03956 |
| Matrix metalloproteinase 13 | MMP13 | P45452 |
| 72 kDa type IV collagenase | MMP2 | P08253 |
| Stromelysin-1 | MMP3 | P08254 |
| Matrix metalloproteinase 9 | MMP9 | P14780 |
| DNA-3-methyladenine glycosylase | MPG | P29372 |
| Myeloperoxidase | MPO | P05164 |
| Myc proto-oncogene protein | MYC | P01106 |
| Myosin light chain kinase, smooth muscle | MYLK | Q15746 |
| Neutrophil cytosol factor 1 | NCF1 | P14598 |
| Nuclear receptor coactivator 1 | NCOA1 | Q15788 |
| Nuclear receptor coactivator 2 | NCOA2 | Q15596 |
| Neocarzinostatin | ncsA | P0A3R9 |
| Serine/threonine-protein kinase NEK2 | NEK2 | P51955 |
| Serine/threonine-protein kinase NEK6 | NEK6 | Q9HC98 |
| Nuclear factor of activated T-cells, cytoplasmic 1 | NFATC1 | O95644 |
| Nuclear factor erythroid 2-related factor 2 | NFE2L2 | Q16236 |
| Nuclear factor NF-kappa-B p105 subunit | NFKB1 | P19838 |
| Nuclear factor NF-kappa-B p100 subunit | NFKB2 | Q00653 |
| NF-kappa-B inhibitor alpha | NFKBIA | P25963 |
| Homeobox protein Nkx-3.1 | NKX3-1 | Q99801 |
| Nitric oxide synthase, inducible | NOS2 | P35228 |
| Nitric oxide synthase, endothelial | NOS3 | P29474 |
| NADPH oxidase 4 | NOX4 | Q9NPH5 |
| NADPH oxidase 5 | NOX5 | Q96PH1 |
| Niemann-Pick C1-like protein 1 | NPC1L1 | Q9UHC9 |
| Puromycin-sensitive aminopeptidase | NPEPPS | P55786 |
| NAD(P)H dehydrogenase [quinone] 1 | NQO1 | P15559 |
| LXR-alpha | NR1H3 | Q13133 |
| Nuclear receptor subfamily 1 group I member 2 | NR1I2 | O75469 |
| Nuclear receptor subfamily 1 group I member 3 | NR1I3 | Q14994 |
| Glucocorticoid receptor | NR3C1 | P04150 |
| Mineralocorticoid receptor | NR3C2 | P08235 |
| NUAK family SNF1-like kinase 1 | NUAK1 | O60285 |
| 26S proteasome non-ATPase regulatory subunit 3 | PSMD3 | O43242 |
| Ornithine decarboxylase | ODC1 | P11926 |
| Delta-type opioid receptor | OPRD1 | P41143 |
| Kappa-type opioid receptor | OPRK1 | P41145 |
| Mu-type opioid receptor | OPRM1 | P35372 |
| Alpha-1-acid glycoprotein 1 | ORM1 | P02763 |
| Poly [ADP-ribose] polymerase 1 | PARP1 | P09874 |
| Procollagen C-endopeptidase enhancer 1 | PCOLCE | Q15113 |
| cAMP and cAMP-inhibited cGMP 3',5'-cyclic phosphodiesterase 10A | PDE10A | Q9Y233 |
| CGMP-inhibited 3',5'-cyclic phosphodiesterase A | PDE3A | Q14432 |
| Heat shock protein HSP 90 | PF3D7_0708400 | Q8IC05 |
| Progesterone receptor | PGR | P06401 |
| Phosphatidylinositol-4,5-bisphosphate 3-kinase catalytic subunit, gamma isoform | PIK3CD | O00329 |
| PI3-kinase p110-gamma subunit | PIK3CG | P48736 |
| PI3-kinase p85-alpha subunit | PIK3R1 | P27986 |
| Serine/threonine-protein kinase PIM1 | PIM1 | P11309 |
| Protein kinase N1 | PKN1 | Q16512 |
| Phospholipase A2 group 1B | PLA2G1B | P04054 |
| Tissue-type plasminogen activator | PLAT | P00750 |
| Urokinase-type plasminogen activator | PLAU | DP00749 |
| Serine/threonine-protein kinase PLK1 | PLK1 | P53350 |
| Serum paraoxonase/arylesterase 1 | PON1 | P27169 |
| NADPH--cytochrome P450 reductase | POR | P16435 |
| Peroxisome proliferator activated receptor alpha | PPARA | Q07869 |
| Peroxisome proliferator-activated receptor delta | PPARD | Q03181 |
| Peroxisome proliferator activated receptor gamma | PPARG | P37231 |
| Serine/threonine-protein phosphatase 2B catalytic subunit alpha isoform | PPP3CA | Q08209 |
| mRNA of PKA Catalytic Subunit C-alpha | PRKACA | P17612 |
| Protein kinase C alpha type | PRKCA | P17252 |
| Protein kinase C beta type | PRKCD | Q05655 |
| Trypsin-1 | PRSS1 | P07477 |
| Tudor domain-containing protein 7 | PRSS10 | P07486 |
| Phosphatidylinositol-3,4,5-trisphosphate 3-phosphatase and dual-specificity protein phosphatase PTEN | PTEN | P60484 |
| Prostaglandin E2 receptor EP3 subtype | PTGER3 | P43115 |
| Prostaglandin G/H synthase 1 | PTGS1 | P23219 |
| Prostaglandin G/H synthase 2 | PTGS2 | P35354 |
| Focal adhesion kinase 1 | PTK2 | Q05397 |
| mRNA of Protein-tyrosine phosphatase, non-receptor type 1 | PTPN1 | P18031 |
| Receptor-type tyrosine-protein phosphatase S | PTPRS | Q13332 |
| Liver glycogen phosphorylase | PYGL | P06737 |
| RAF proto-oncogene serine/threonine-protein kinase | RAF1 | P04049 |
| Ras GTPase-activating protein 1 | RASA1 | P20936 |
| Ras association domain-containing protein 1 | RASSF1 | Q9NS23 |
| Retinoblastoma-associated protein | RB1 | P06400 |
| Transcription factor p65 | RELA | Q04206 |
| Nuclear receptor ROR-alpha | RORA | P11473 |
| Nuclear receptor ROR-gamma | RORC | P51449 |
| Protein CBFA2T1 | RUNX1T1 | Q06455 |
| Runt-related transcription factor 2 | RUNX2 | Q13950 |
| Retinoic acid receptor RXR-alpha | RXRA | P19793 |
| Retinoic acid receptor RXR-beta | RXRB | P28702 |
| SUMO-activating enzyme subunit 1 | SAE1 | Q9UBE0 |
| Sodium channel protein type 5 subunit alpha | SCN1A | P35498 |
| E-selectin | SELE | P16581 |
| Plasminogen activator inhibitor 1 | SERPINE1 | P05121 |
| Sex hormone-binding globulin | SHBG | P04278 |
| Sigma non-opioid intracellular receptor 1 | SIGMAR1 | Q99720 |
| Synaptic vesicular amine transporter | SLC18A2 | Q05940 |
| Solute carrier family 22 member 12 | SLC22A12 | Q96S37 |
| Solute carrier family 2, facilitated glucose transporter member 4 | SLC2A4 | P14672 |
| Sodium-dependent noradrenaline transporter | SLC6A2 | P23975 |
| Sodium-dependent dopamine transporter | SLC6A3 | Q01959 |
| Sodium-dependent serotonin transporter | SLC6A4 | P31645 |
| Antileukoproteinase | SLPI | P03973 |
| Superoxide dismutase [Cu-Zn] | SOD1 | P00441 |
| Osteopontin | SPP1 | P10451 |
| Tyrosine-protein kinase SRC | SRC | P12931 |
| 3-oxo-5-alpha-steroid 4-dehydrogenase 1 | SRD5A1 | P18405 |
| Signal transducer and activator of transcription 1-alpha/beta | STAT1 | P42224 |
| Estrogen sulfotransferase | SULT1E1 | P49888 |
| Bile salt sulfotransferase | SULT2A1 | Q06520 |
| Sulfotransferase family cytosolic 2B member 1 | SULT2B1 | O00204 |
| Tyrosine-protein kinase SYK | SYK | P43405 |
| Telomerase protein component 1 | TEP1 | Q99973 |
| Transforming growth factor beta-1 | TGFB1 | P01137 |
| Thrombomodulin | THBD | P07204 |
| Tumor necrosis factor | TNF | P01375 |
| DNA topoisomerase II alpha | TOP2A | P11388 |
| DNA topoisomerase II beta | TOP2B | Q02880 |
| DNA topoisomerase 1 | topA | P06612 |
| Cellular tumor antigen p53 | TP53 | P04637 |
| Tyrosinase | TYR | P14679 |
| SUMO-activating enzyme subunit 2 | UBA2 | Q9UBT2 |
| Vascular cell adhesion protein 1 | VCAM1 | P19320 |
| Vitamin D3 receptor | VDR | P11473 |
| Vascular endothelial growth factor A | VEGFA | P15692 |
| Xanthine dehydrogenase | XDH | P47989 |

**Supplementary Table 3 The topological parameters of herb-compound-target network**

| **name** | **Degree** | **name** | **Degree** |
| --- | --- | --- | --- |
| CZ | 8 | AXL | 1 |
| HB | 29 | BACE1 | 1 |
| NX | 19 | BAD | 1 |
| ABCB1 | 2 | BAX | 5 |
| ABCC1 | 2 | BCHE | 3 |
| ABCG2 | 2 | BCL2 | 5 |
| ACACA | 1 | BCL2L1 | 1 |
| ACHE | 10 | BIRC5 | 8 |
| ACPP | 1 | CA1 | 1 |
| ADH1C | 1 | CA12 | 2 |
| ADORA1 | 2 | CA13 | 1 |
| ADORA2A | 2 | CA14 | 1 |
| ADRA1A | 6 | CA2 | 3 |
| ADRA1B | 8 | CA3 | 1 |
| ADRA1D | 4 | CA4 | 2 |
| ADRA2A | 1 | CA5A | 1 |
| ADRA2B | 3 | CA6 | 1 |
| ADRA2C | 4 | CA7 | 2 |
| ADRB1 | 2 | CA9 | 1 |
| ADRB2 | 13 | CACNA1S | 1 |
| AHR | 4 | CALM1 | 12 |
| AHSA1 | 3 | CAMK2B | 1 |
| AKR1A1 | 1 | CASP3 | 6 |
| AKR1B1 | 3 | CASP8 | 2 |
| AKR1C1 | 1 | CASP9 | 3 |
| AKR1C2 | 1 | CAV1 | 1 |
| AKR1C3 | 3 | CBR1 | 1 |
| AKR1C4 | 1 | CCL2 | 2 |
| AKT1 | 4 | CCNB1 | 2 |
| ALK | 1 | CCND1 | 2 |
| ALOX12 | 3 | CD40LG | 1 |
| ALOX15 | 3 | CDK1 | 3 |
| ALOX5 | 2 | CDK2 | 3 |
| ANXA1 | 3 | CDK5R1 | 2 |
| APEX1 | 1 | CDK6 | 1 |
| APOD | 1 | CDKN1A | 2 |
| AR | 32 | CDKN2A | 1 |
| ARG1 | 2 | CHEK1 | 3 |
| AURKB | 1 | CHEK2 | 1 |
| AVPR2 | 1 | choB | 15 |
| CHRM1 | 21 | DUOX2 | 1 |
| CHRM2 | 7 | E2F1 | 1 |
| CHRM3 | 8 | E2F2 | 1 |
| CHRM4 | 6 | EGF | 1 |
| CHRM5 | 5 | EGFR | 1 |
| CHRNA2 | 4 | EGLN1 | 1 |
| CHRNA7 | 7 | EIF6 | 2 |
| CHUK | 1 | ELK1 | 1 |
| CLDN4 | 1 | ERBB2 | 1 |
| CLEC4E | 13 | ERBB3 | 1 |
| COL1A1 | 1 | ESR1 | 28 |
| COL3A1 | 1 | ESR2 | 20 |
| CRK2 | 3 | ESRRA | 4 |
| CRP | 1 | ESRRB | 4 |
| CSNK2A1 | 1 | F10 | 10 |
| CTRB1 | 1 | F2 | 6 |
| CTSD | 1 | F3 | 4 |
| CXCL10 | 1 | F7 | 8 |
| CXCL11 | 1 | FABP5 | 1 |
| CXCL2 | 1 | FLT3 | 2 |
| CXCL8 | 2 | FN1 | 1 |
| CXCR1 | 1 | FOS | 2 |
| CYCS | 1 | FOSL1 | 1 |
| CYP17A1 | 6 | FOSL2 | 1 |
| CYP19A1 | 1 | GABR(Protein Group) | 15 |
| CYP1A1 | 3 | GABRA1 | 9 |
| CYP1A2 | 3 | GABRA2 | 2 |
| CYP1B1 | 4 | GABRA6 | 1 |
| CYP2B6 | 1 | GABRB3 | 1 |
| CYP2D6 | 1 | GJA1 | 1 |
| CYP3A4 | 2 | GLO1 | 2 |
| CYP3A5 | 1 | GPR35 | 2 |
| CYP51A1 | 5 | GRIA2 | 1 |
| DAPK1 | 2 | GRIN(Protein Group) | 15 |
| DCAF1 | 1 | GRK6 | 1 |
| DIO1 | 2 | GSK3B | 4 |
| DNA | 1 | GSTM1 | 2 |
| DPP4 | 7 | GSTM2 | 2 |
| DRD1 | 7 | GSTP1 | 2 |
| DRD2 | 2 | gyrB | 1 |
| DRD3 | 2 | HAS2 | 2 |
| DRD4 | 2 | HDAC2 | 1 |
| DRD5 | 2 | HERC5 | 1 |
| HIF1AN | 2 | MAOA | 3 |
| HK2 | 1 | MAOB | 2 |
| HMGCR | 7 | MAP2 | 1 |
| HMOX1 | 2 | MAPK1 | 1 |
| HSD11B1 | 1 | MAPK14 | 1 |
| HSD11B2 | 3 | MAPK8 | 1 |
| HSD17B1 | 16 | MAPK8IP1 | 1 |
| HSD17B2 | 2 | MAPT | 1 |
| HSD3B1 | 3 | MCL1 | 1 |
| HSF1 | 1 | MET | 1 |
| HSPA5 | 1 | MGAM | 1 |
| HSPB1 | 1 | MMP1 | 3 |
| HTR1A | 1 | MMP13 | 1 |
| HTR2A | 8 | MMP2 | 3 |
| HTR2B | 1 | MMP3 | 1 |
| HTR2C | 3 | MMP9 | 4 |
| HTR3A | 5 | MPG | 2 |
| HTR7 | 1 | MPO | 2 |
| ICAM1 | 2 | MYC | 1 |
| IFNG | 1 | MYLK | 1 |
| IGF1R | 1 | NCF1 | 1 |
| IGF2 | 2 | NCOA1 | 4 |
| IGFBP3 | 1 | NCOA2 | 15 |
| IGHG1 | 2 | ncsA | 4 |
| IKBKB | 1 | NEK2 | 1 |
| IL10 | 1 | NEK6 | 1 |
| IL1A | 1 | NFATC1 | 1 |
| IL1B | 1 | NFE2L2 | 1 |
| IL2 | 1 | NFKB1 | 1 |
| IL4 | 1 | NFKB2 | 1 |
| IL6 | 2 | NFKBIA | 1 |
| INSR | 2 | NKX3-1 | 1 |
| IRF1 | 1 | NOS2 | 9 |
| JUN | 4 | NOS3 | 10 |
| KCNH2 | 13 | NOX4 | 2 |
| KCNMA1 | 1 | NOX5 | 1 |
| KDM4E | 2 | NPC1L1 | 9 |
| KDR | 3 | NPEPPS | 1 |
| KRT12 | 1 | NQO1 | 1 |
| KRT2 | 1 | NR1H3 | 9 |
| ksi | 15 | NR1I2 | 17 |
| LKHA4 | 1 | NR1I3 | 17 |
| LPL | 1 | NR3C1 | 6 |
| NR3C2 | 8 | RASA1 | 1 |
| NUAK1 | 1 | RASSF1 | 1 |
| ODC1 | 1 | RB1 | 1 |
| OPRD1 | 5 | RELA | 4 |
| OPRK1 | 1 | RORA | 13 |
| OPRM1 | 7 | RORC | 9 |
| ORM1 | 1 | RUNX1T1 | 1 |
| PARP1 | 1 | RUNX2 | 1 |
| PCOLCE | 1 | RXRA | 16 |
| PDE10A | 3 | RXRB | 3 |
| PDE3A | 6 | SAE1 | 1 |
| PF3D7_0708400 | 16 | SCN1A | 17 |
| PGR | 12 | SELE | 2 |
| PIK3CD | 7 | SERPINE1 | 1 |
| PIK3CG | 1 | SHBG | 2 |
| PIK3R1 | 1 | SIGMAR1 | 17 |
| PIM1 | 4 | SLC18A2 | 2 |
| PKN1 | 1 | SLC22A12 | 2 |
| PLA2G1B | 1 | SLC2A4 | 2 |
| PLAT | 1 | SLC6A2 | 4 |
| PLAU | 2 | SLC6A3 | 5 |
| PLK1 | 1 | SLC6A4 | 6 |
| PON1 | 2 | SLPI | 1 |
| POR | 1 | SOD1 | 1 |
| PPARA | 16 | SPP1 | 1 |
| PPARD | 1 | SRC | 1 |
| PPARG | 4 | SRD5A1 | 1 |
| PPP3CA | 1 | STAT1 | 2 |
| PRKACA | 1 | SULT1E1 | 1 |
| PRKCA | 3 | SULT2A1 | 15 |
| PRKCD | 3 | SULT2B1 | 15 |
| PRSS1 | 10 | SYK | 2 |
| PRSS10 | 1 | TEP1 | 1 |
| PSMD3 | 2 | TGFB1 | 2 |
| PTEN | 1 | THBD | 1 |
| PTGER3 | 2 | TNF | 5 |
| PTGS1 | 19 | TOP2A | 6 |
| PTGS2 | 25 | TOP2B | 6 |
| PTK2 | 1 | topA | 1 |
| PTPN1 | 2 | TP53 | 3 |
| PTPRS | 2 | TYR | 1 |
| PYGL | 1 | UBA2 | 1 |
| RAF1 | 1 | VCAM1 | 2 |
| VDR | 13 | MOL002672 | 2 |
| VEGFA | 2 | MOL002671 | 3 |
| XDH | 3 | MOL000762 | 4 |
| MOL005438 | 25 | MOL002641 | 4 |
| MOL000085 | 26 | MOL000622 | 5 |
| MOL000787 | 28 | MOL002663 | 6 |
| MOL001454 | 28 | MOL002776 | 6 |
| MOL002670 | 35 | MOL002666 | 7 |
| MOL001455 | 40 | MOL012461 | 8 |
| MOL000790 | 46 | MOL012537 | 8 |
| MOL002714 | 47 | MOL002668 | 9 |
| MOL000173 | 51 | MOL000184 | 11 |
| MOL000449 | 52 | MOL003847 | 11 |
| MOL000358 | 56 | MOL001131 | 12 |
| MOL000422 | 97 | MOL001458 | 12 |
| MOL000098 | 226 | MOL002644 | 14 |
| MOL001006 | 21 | MOL002894 | 15 |
| MOL002643 | 21 | MOL002897 | 16 |
| MOL004355 | 21 | MOL006422 | 16 |
| MOL000092 | 22 | MOL002660 | 19 |
| MOL000188 | 22 | MOL002662 | 19 |
| MOL002651 | 22 | MOL006413 | 20 |
| MOL000785 | 23 | MOL000088 | 24 |
| MOL001771 | 23 | MOL000094 | 24 |

**Supplementary Table 4 GA related targets**

| **Gene_Full_Name** | **Gene** | **Source** |
| --- | --- | --- |
| ATP Binding Cassette Subfamily G Member 2 | ABCG2 | GeneCards |
| Adenosine Deaminase | ADA | GeneCards |
| ADAM metallopeptidase with thrombospondin type 1 motif 3 | ADAMTS3 | DisGeNet |
| ADAM metallopeptidase with thrombospondin type 1 motif 4 | ADAMTS4 | DisGeNet |
| Adenylosuccinate Lyase | ADSL | GeneCards |
| Acylglycerol Kinase | AGK | GeneCards |
| Aldehyde Dehydrogenase 2 Family Member | ALDH2 | GeneCards |
| Alpha Kinase 1 | ALPK1 | GeneCards |
| Adenosine Monophosphate Deaminase 3 | AMPD3 | GeneCards |
| Apolipoprotein A1 | APOA1 | GeneCards, DisGeNet |
| Apolipoprotein A2 | APOA2 | GeneCards |
| Apolipoprotein C2 | APOC2 | GeneCards |
| Apolipoprotein E | APOE | GeneCards |
| Amyloid Beta Precursor Protein | APP | GeneCards |
| Adenine Phosphoribosyltransferase | APRT | GeneCards |
| Rho GTPase Activating Protein 26 | ARHGAP26 | GeneCards |
| atrophin 1 | ATN1 | DisGeNet |
| branched chain keto acid dehydrogenase E1, alpha polypeptide | BCKDHA | DisGeNet |
| Bone Morphogenetic Protein 2 | BMP2 | GeneCards |
| Caspase Recruitment Domain Family Member 8 | CARD8 | GeneCards, DisGeNet |
| Capping Protein Regulator And Myosin 1 Linker 1 | CARMIL1 | GeneCards |
| Caspase 1 | CASP1 | GeneCards |
| C-C Motif Chemokine Ligand 2 | CCL2 | GeneCards |
| C-C Motif Chemokine Ligand 20 | CCL20 | GeneCards |
| CD14 Molecule | CD14 | GeneCards |
| Choline Kinase Beta | CHKB | GeneCards |
| C-Type Lectin Domain Family 12 Member A | CLEC12A | GeneCards |
| Complement C3b/C4b Receptor 1 (Knops Blood Group) | CR1 | GeneCards |
| C-Reactive Protein | CRP | GeneCards, DisGeNet |
| Colony Stimulating Factor 1 | CSF1 | GeneCards |
| Colony Stimulating Factor 2 | CSF2 | GeneCards |
| Cystatin C | CST3 | GeneCards |
| Cathepsin B | CTSB | GeneCards |
| C-X-C Motif Chemokine Ligand 2 | CXCL2 | GeneCards |
| C-X-C Motif Chemokine Ligand 8 | CXCL8 | GeneCards, DisGeNet |
| Aspartyl-TRNA Synthetase 2, Mitochondrial | DARS2 | GeneCards |
| Delta Like Canonical Notch Ligand 1 | DLL1 | GeneCards |
| EPH Receptor A2 | EPHA2 | GeneCards |
| Coagulation Factor II, Thrombin | F2 | GeneCards |
| Glucose-6-Phosphatase Catalytic Subunit | G6PC | GeneCards, DisGeNet |
| Glucose-6-Phosphatase Catalytic Subunit 3 | G6PC3 | GeneCards |
| Glutamate Decarboxylase 1 | GAD1 | GeneCards |
| Galactokinase 1 | GALK1 | GeneCards |
| Major Histocompatibility Complex, Class I, B | HLA-B | GeneCards |
| Heme Oxygenase 1 | HMOX1 | GeneCards |
| HNF1 Homeobox B | HNF1B | GeneCards, DisGeNet |
| Hyperuricemic Nephropathy, Familial Juvenile, 3 | HNFJ3 | GeneCards |
| Haptoglobin | HP | GeneCards |
| Hypoxanthine Phosphoribosyltransferase 1 | HPRT1 | GeneCards, DisGeNet |
| 5-Hydroxytryptamine Receptor 3A | HTR3A | GeneCards |
| Interleukin 16 | IL16 | GeneCards |
| Interleukin 17A | IL17A | GeneCards |
| interleukin 18 | IL18 | GeneCards, DisGeNet |
| Interleukin 1 Beta | IL1B | GeneCards, DisGeNet |
| Interleukin 1 Receptor Accessory Protein Like 2 | IL1RAPL2 | GeneCards |
| Interleukin 1 Receptor Antagonist | IL1RN | GeneCards |
| Interleukin 6 | IL6 | GeneCards, DisGeNet |
| Interleukin 6 Receptor | IL6R | GeneCards |
| Inositol Polyphosphate-5-Phosphatase D | INPP5D | GeneCards, DisGeNet |
| Insulin | INS | GeneCards |
| Interleukin 1 Receptor Associated Kinase 4 | IRAK4 | GeneCards |
| Killer Cell Lectin Like Receptor G1 | KLRG1 | GeneCards |
| Long Intergenic Non-Protein Coding RNA 1428 | LINC01428 | GeneCards |
| Lipoprotein(A) | LPA | GeneCards |
| Lipoprotein Lipase | LPL | GeneCards |
| Mitogen-Activated Protein Kinase 1 | MAPK1 | GeneCards |
| Mitogen-Activated Protein Kinase 14 | MAPK14 | GeneCards |
| Melanocortin 1 Receptor | MC1R | GeneCards |
| Melanocortin 3 Receptor | MC3R | GeneCards |
| Melanocortin 5 Receptor | MC5R | GeneCards |
| Medullary Cystic Kidney Disease 2 (Autosomal Dominant) | MCKD2 | GeneCards |
| MEFV Innate Immuity Regulator, Pyrin | MEFV | GeneCards |
| Methyltransferase Like 6 | METTL6 | GeneCards |
| MicroRNA 146a | MIR146A | GeneCards |
| MicroRNA 155 | MIR155 | GeneCards, DisGeNet |
| MicroRNA 302b | MIR302B | GeneCards |
| MicroRNA 488 | MIR488 | GeneCards, DisGeNet |
| MicroRNA 920 | MIR920 | GeneCards |
| Matrix Metallopeptidase 2 | MMP2 | GeneCards |
| Matrix Metallopeptidase 3 | MMP3 | GeneCards |
| Matrix Metallopeptidase 9 | MMP9 | GeneCards, DisGeNet |
| Mucin 1, Cell Surface Associated | MUC1 | GeneCards, DisGeNet |
| MYD88 Innate Immune Signal Transduction Adaptor | MYD88 | GeneCards |
| Myosin Heavy Chain 9 | MYH9 | GeneCards |
| Myosin IXA | MYO9A | GeneCards |
| Nuclear Factor, Erythroid 2 Like 2 | NFE2L2 | GeneCards |
| nuclear factor kappa B subunit 1 | NFKB1 | DisGeNet |
| Nerve Growth Factor | NGF | GeneCards |
| NLR Family Pyrin Domain Containing 3 | NLRP3 | GeneCards, DisGeNet |
| Nucleotide Binding Oligomerization Domain Containing 2 | NOD2 | GeneCards |
| Nitric Oxide Synthase 2 | NOS2 | GeneCards |
| Nephrocystin 1 | NPHP1 | GeneCards |
| Purinergic Receptor P2X7 | P2RX7 | GeneCards, DisGeNet |
| Phosphofructokinase, Muscle | PFKM | GeneCards,DisGeNet |
| Phosphorylase Kinase Regulatory Subunit Alpha 2 | PHKA2 | GeneCards |
| Phosphatidylinositol-4,5-Bisphosphate 3-Kinase Catalytic Subunit Gamma | PIK3CG | GeneCards |
| Phospholipase A2 Group IVA | PLA2G4A | GeneCards |
| Phospholipase A2 Activating Protein | PLAA | GeneCards |
| Plasminogen Activator, Urokinase | PLAU | GeneCards |
| Purine Nucleoside Phosphorylase | PNP | GeneCards |
| Proopiomelanocortin | POMC | GeneCards,DisGeNet |
| Peroxisome Proliferator Activated Receptor Gamma | PPARG | GeneCards |
| PPARG Coactivator 1 Beta | PPARGC1B | GeneCards |
| Phosphoribosyl Pyrophosphate Amidotransferase | PPAT | GeneCards |
| Phosphoribosyl Pyrophosphate Synthetase 1 | PRPS1 | GeneCards,DisGeNet |
| Prostaglandin-Endoperoxide Synthase 1 | PTGS1 | GeneCards |
| Prostaglandin-Endoperoxide Synthase 2 | PTGS2 | GeneCards |
| Rap Guanine Nucleotide Exchange Factor 3 | RAPGEF3 | GeneCards |
| Rap Guanine Nucleotide Exchange Factor 4 | RAPGEF4 | GeneCards |
| RELA Proto-Oncogene, NF-KB Subunit | RELA | GeneCards |
| Renin | REN | GeneCards |
| S100 Calcium Binding Protein A8 | S100A8 | GeneCards |
| S100 Calcium Binding Protein A9 | S100A9 | GeneCards |
| SEC61 Translocon Alpha 1 Subunit | SEC61A1 | GeneCards,DisGeNet |
| Serpin Family E Member 1 | SERPINE1 | GeneCards |
| Solute Carrier Family 10 Member 2 | SLC10A2 | GeneCards |
| Solute Carrier Family 16 Member 9 | SLC16A9 | GeneCards |
| Solute Carrier Family 17 Member 1 | SLC17A1 | GeneCards,DisGeNet |
| Solute Carrier Family 17 Member 3 | SLC17A3 | GeneCards |
| Solute Carrier Family 17 Member 5 | SLC17A5 | GeneCards |
| Solute Carrier Family 22 Member 11 | SLC22A11 | GeneCards,DisGeNet |
| Solute Carrier Family 22 Member 12 | SLC22A12 | GeneCards,DisGeNet |
| Solute Carrier Family 22 Member 6 | SLC22A6 | GeneCards |
| solute carrier family 22 member 9 | SLC22A9 | DisGeNet |
| Solute Carrier Family 2 Member 6 | SLC2A6 | GeneCards |
| Solute Carrier Family 2 Member 9 | SLC2A9 | GeneCards |
| solute carrier family 2 member 9 | SLC2A9 | DisGeNet |
| Solute Carrier Family 37 Member 4 | SLC37A4 | GeneCards,DisGeNet |
| SRC Proto-Oncogene, Non-Receptor Tyrosine Kinase | SRC | GeneCards |
| Spleen Associated Tyrosine Kinase | SYK | GeneCards |
| Tec Protein Tyrosine Kinase | TEC | GeneCards |
| Telomerase Reverse Transcriptase | TERT | GeneCards |
| Transforming Growth Factor Beta 1 | TGFB1 | GeneCards |
| TIMP Metallopeptidase Inhibitor 1 | TIMP1 | GeneCards |
| Toll Like Receptor 2 | TLR2 | GeneCards |
| Toll Like Receptor 4 | TLR4 | GeneCards,DisGeNet |
| Toll Like Receptor 5 | TLR5 | GeneCards |
| Tumor Necrosis Factor | TNF | GeneCards |
| TNF Superfamily Member 11 | TNFSF11 | GeneCards,DisGeNet |
| Triggering Receptor Expressed On Myeloid Cells 1 | TREM1 | GeneCards,DisGeNet |
| Uromodulin | UMOD | GeneCards,DisGeNet |
| Urate Oxidase (Pseudogene) | UOX | GeneCards,DisGeNet |
| Vitamin D Receptor | VDR | GeneCards |
| WD Repeat Domain 1 | WDR1 | GeneCards |
| Xanthine Dehydrogenase | XDH | GeneCards |

**Supplementary Table 5 The topological parameters of compound-target network**

| **name** | **Degree** | **name** | **Degree** |
| --- | --- | --- | --- |
| PTGS2 | 25 | MOL000449 | 4 |
| PTGS1 | 19 | MOL000188 | 2 |
| VDR | 13 | MOL002644 | 2 |
| NOS2 | 9 | MOL002666 | 2 |
| F2 | 6 | MOL002897 | 2 |
| HTR3A | 5 | MOL003847 | 2 |
| TNF | 5 | MOL000785 | 3 |
| MMP9 | 4 | MOL000787 | 3 |
| PPARG | 4 | MOL000790 | 3 |
| RELA | 4 | MOL001454 | 3 |
| MMP2 | 3 | MOL001455 | 3 |
| XDH | 3 | MOL001458 | 3 |
| ABCG2 | 2 | MOL002651 | 3 |
| CCL2 | 2 | MOL002668 | 3 |
| CXCL8 | 2 | MOL002670 | 3 |
| HMOX1 | 2 | MOL002894 | 3 |
| IL6 | 2 | MOL006422 | 3 |
| PLAU | 2 | MOL012537 | 3 |
| SLC22A12 | 2 | MOL000085 | 1 |
| SYK | 2 | MOL000088 | 1 |
| TGFB1 | 2 | MOL000092 | 1 |
| CRP | 1 | MOL000094 | 1 |
| CXCL2 | 1 | MOL000186 | 1 |
| IL1B | 1 | MOL001006 | 1 |
| LPL | 1 | MOL001131 | 1 |
| MAPK1 | 1 | MOL001771 | 1 |
| MAPK14 | 1 | MOL002641 | 1 |
| MMP3 | 1 | MOL002643 | 1 |
| NFE2L2 | 1 | MOL002672 | 1 |
| NFKB1 | 1 | MOL004355 | 1 |
| PIK3CG | 1 | MOL005438 | 1 |
| SERPINE1 | 1 | MOL012461 | 1 |
| SRC | 1 | MOL000358 | 4 |
| MOL000098 | 27 | MOL002662 | 6 |
| MOL000422 | 14 | MOL002714 | 5 |
| MOL000173 | 10 |  |  |

**Supplementary Table 6 the topological parameters of protein-protein network**

| **name** | **Degree** |
| --- | --- |
| TNF | 28 |
| IL6 | 28 |
| CXCL8 | 27 |
| CCL2 | 25 |
| IL1B | 24 |
| SRC | 24 |
| PTGS2 | 24 |
| MAPK1 | 23 |
| PPARG | 23 |
| TGFB1 | 22 |
| MMP9 | 22 |
| RELA | 19 |
| MAPK14 | 19 |
| SERPINE1 | 18 |
| HMOX1 | 18 |
| NFKB1 | 18 |
| MMP2 | 18 |
| CRP | 17 |
| NOS2 | 16 |
| MMP3 | 16 |
| PLAU | 14 |
| CXCL2 | 12 |
| F2 | 12 |
| SYK | 11 |
| NFE2L2 | 10 |
| VDR | 10 |
| PTGS1 | 8 |
| LPL | 7 |
| PIK3CG | 5 |
| ABCG2 | 5 |
| XDH | 3 |
| SLC22A12 | 2 |

**Supplementary Table 7 The results of GOBP enrichment analysis**

| **Term** | **Name** | **PValue** |
| --- | --- | --- |
| GO:0031663 | lipopolysaccharide-mediated signaling pathway | 0.000000000110 |
| GO:0045893 | positive regulation of transcription, DNA-templated | 0.000000014131 |
| GO:0051092 | positive regulation of NF-kappaB transcription factor activity | 0.000000165703 |
| GO:0071347 | cellular response to interleukin-1 | 0.000000669871 |
| GO:0045944 | positive regulation of transcription from RNA polymerase II promoter | 0.000001996955 |
| GO:0043491 | protein kinase B signaling | 0.000004614804 |
| GO:0010628 | positive regulation of gene expression | 0.000004724270 |
| GO:0043065 | positive regulation of apoptotic process | 0.000009133378 |
| GO:0031622 | positive regulation of fever generation | 0.000009629721 |
| GO:0042493 | response to drug | 0.000009739573 |
| GO:0045429 | positive regulation of nitric oxide biosynthetic process | 0.000010373176 |
| GO:0070374 | positive regulation of ERK1 and ERK2 cascade | 0.000024398997 |
| GO:0071316 | cellular response to nicotine | 0.000026915077 |
| GO:0010888 | negative regulation of lipid storage | 0.000026915077 |
| GO:0071407 | cellular response to organic cyclic compound | 0.000027053077 |
| GO:0048661 | positive regulation of smooth muscle cell proliferation | 0.000028459108 |
| GO:0071260 | cellular response to mechanical stimulus | 0.000047206935 |
| GO:0051897 | positive regulation of protein kinase B signaling | 0.000078061709 |
| GO:0000165 | MAPK cascade | 0.000117461265 |
| GO:0031293 | membrane protein intracellular domain proteolysis | 0.000146198340 |
| GO:0051091 | positive regulation of sequence-specific DNA binding transcription factor activity | 0.000151571741 |
| GO:0000187 | activation of MAPK activity | 0.000160285275 |
| GO:0071356 | cellular response to tumor necrosis factor | 0.000173957066 |
| GO:0071375 | cellular response to peptide hormone stimulus | 0.000181337402 |
| GO:0042346 | positive regulation of NF-kappaB import into nucleus | 0.000200306242 |
| GO:0001934 | positive regulation of protein phosphorylation | 0.000265865362 |
| GO:0019221 | cytokine-mediated signaling pathway | 0.000291269050 |
| GO:0010575 | positive regulation of vascular endothelial growth factor production | 0.000333603709 |
| GO:0071549 | cellular response to dexamethasone stimulus | 0.000385418632 |
| GO:0043200 | response to amino acid | 0.000440902513 |
| GO:0008285 | negative regulation of cell proliferation | 0.000568237085 |
| GO:0007568 | aging | 0.000572480705 |
| GO:0006955 | immune response | 0.000715286935 |
| GO:0045599 | negative regulation of fat cell differentiation | 0.000811053603 |
| GO:0032755 | positive regulation of interleukin-6 production | 0.000930907106 |
| GO:0043066 | negative regulation of apoptotic process | 9.56E-04 |
| GO:0043406 | positive regulation of MAP kinase activity | 0.001595527 |
| GO:0051384 | response to glucocorticoid | 0.001932716 |
| GO:0033138 | positive regulation of peptidyl-serine phosphorylation | 0.002237333 |
| GO:0050731 | positive regulation of peptidyl-tyrosine phosphorylation | 0.003054812 |
| GO:0050830 | defense response to Gram-positive bacterium | 0.003278004 |
| GO:0002223 | stimulatory C-type lectin receptor signaling pathway | 0.004954204 |
| GO:0000122 | negative regulation of transcription from RNA polymerase II promoter | 0.005102505 |
| GO:0010629 | negative regulation of gene expression | 0.008293952 |

**Supplementary Table 8 The results of KEGG pathway enrichment analysis**

| **Term** | **Name** | **PValue** | **Genes** |
| --- | --- | --- | --- |
| hsa05410 | Hypertrophic cardiomyopathy (HCM) | 0.0137423190960840 | P01375, P05231, P01137 |
| hsa05332 | Graft-versus-host disease | 0.0025679725127165 | P01375, P05231, P01584 |
| hsa05323 | Rheumatoid arthritis | 0.0000011945782265 | P13500, P01375, P05231, P10145, P01137, P01584 |
| hsa05321 | Inflammatory bowel disease (IBD) | 0.0000002401058793 | Q04206, P01375, P19838, P05231, P01137, P01584 |
| hsa05221 | Acute myeloid leukemia | 0.0072601805561550 | P28482, Q04206, P19838 |
| hsa05220 | Chronic myeloid leukemia | 0.0005583921375695 | P28482, Q04206, P19838, P01137 |
| hsa05219 | Bladder cancer | 0.0000018774225380 | P12931, P08253, P28482, P14780, P10145 |
| hsa05215 | Prostate cancer | 0.0172826160970196 | P28482, Q04206, P19838 |
| hsa05212 | Pancreatic cancer | 0.0004130509582640 | P28482, Q04206, P19838, P01137 |
| hsa05205 | Proteoglycans in cancer | 0.0000035192618716 | P12931, P08253, P28482, P01375, P14780, Q16539, P01137 |
| hsa05203 | Viral carcinogenesis | 0.0109641883365616 | P12931, P28482, Q04206, P19838 |
| hsa05169 | Epstein-Barr virus infection | 0.0318299490974536 | Q04206, Q16539, P19838 |
| hsa05168 | Herpes simplex infection | 0.0000434179813169 | P13500, Q04206, P01375, P19838, P05231, P01584 |
| hsa05166 | HTLV-I infection | 0.0023254882699684 | Q04206, P01375, P19838, P05231, P01137 |
| hsa05164 | Influenza A | 0.0000000015461073 | P13500, P28482, Q04206, P01375, Q16539, P19838, P05231, P10145, P01584 |
| hsa05162 | Measles | 0.0032906833255004 | Q04206, P19838, P05231, P01584 |
| hsa05161 | Hepatitis B | 0.0000000003586177 | P12931, P28482, Q04206, P01375, P14780, P19838, P05231, P10145, P01137 |
| hsa05160 | Hepatitis C | 0.0000092242398609 | P28482, Q04206, P01375, Q16539, P19838, P10145 |
| hsa05152 | Tuberculosis | 0.0000000017720461 | P12931, P28482, Q04206, P01375, Q16539, P19838, P05231, P01137, P01584 |
| hsa05146 | Amoebiasis | 0.0000000820613557 | Q04206, P01375, P19838, P05231, P10145, P01137, P01584 |
| hsa05145 | Toxoplasmosis | 0.0000036232302496 | P28482, Q04206, P01375, Q16539, P19838, P01137 |
| hsa05144 | Malaria | 0.0000000612697787 | P13500, P01375, P05231, P10145, P01137, P01584 |
| hsa05143 | African trypanosomiasis | 0.0025679725127165 | P01375, P05231, P01584 |
| hsa05142 | Chagas disease (American trypanosomiasis) | 0.0000000000000030 | P13500, P05121, P28482, Q04206, P01375, Q16539, P19838, P05231, P10145, P01137, P01584 |
| hsa05140 | Leishmaniasis | 0.0000000000980257 | P35354, P28482, Q04206, P01375, Q16539, P19838, P01137, P01584 |
| hsa05134 | Legionellosis | 0.0000001009356920 | Q04206, P01375, P19838, P05231, P10145, P01584 |
| hsa05133 | Pertussis | 0.0000000001456265 | P28482, Q04206, P01375, Q16539, P19838, P05231, P10145, P01584 |
| hsa05132 | Salmonella infection | 0.0000000186903140 | P28482, Q04206, Q16539, P19838, P05231, P10145, P01584 |
| hsa05131 | Shigellosis | 0.0000002401058793 | P12931, P28482, Q04206, Q16539, P19838, P10145 |
| hsa05120 | Epithelial cell signaling in Helicobacter pylori infection | 0.0000137010880023 | P12931, Q04206, Q16539, P19838, P10145 |
| hsa04932 | Non-alcoholic fatty liver disease (NAFLD) | 0.0000006766229227 | Q04206, P01375, P19838, P05231, P10145, P01137, P01584 |
| hsa04931 | Insulin resistance | 0.0018161088950174 | Q04206, P01375, P19838, P05231 |
| hsa04920 | Adipocytokine signaling pathway | 0.0111716800947178 | Q04206, P01375, P19838 |
| hsa04917 | Prolactin signaling pathway | 0.0000172711085548 | P12931, P28482, Q04206, Q16539, P19838 |
| hsa04915 | Estrogen signaling pathway | 0.0014132474151973 | P12931, P08253, P28482, P14780 |
| hsa04912 | GnRH signaling pathway | 0.0011071277312262 | P12931, P08253, P28482, Q16539 |
| hsa04722 | Neurotrophin signaling pathway | 0.0024560011247859 | P28482, Q04206, Q16539, P19838 |
| hsa04668 | TNF signaling pathway | 0.0000000000003963 | P13500, P35354, P28482, Q04206, P01375, P14780, Q16539, P19838, P05231, P01584 |
| hsa04664 | Fc epsilon RI signaling pathway | 0.0105664632085295 | P28482, P01375, Q16539 |
| hsa04662 | B cell receptor signaling pathway | 0.0108671741879285 | P28482, Q04206, P19838 |
| hsa04660 | T cell receptor signaling pathway | 0.0000669193371030 | P28482, Q04206, P01375, Q16539, P19838 |
| hsa04640 | Hematopoietic cell lineage | 0.0169126550275788 | P01375, P05231, P01584 |
| hsa04623 | Cytosolic DNA-sensing pathway | 0.0003945472055937 | Q04206, P19838, P05231, P01584 |
| hsa04622 | RIG-I-like receptor signaling pathway | 0.0000163209718245 | Q04206, P01375, Q16539, P19838, P10145 |
| hsa04621 | NOD-like receptor signaling pathway | 0.0000000000001404 | P13500, P28482, Q04206, P01375, Q16539, P19838, P05231, P10145, P01584 |
| hsa04620 | Toll-like receptor signaling pathway | 0.0000000017250180 | P28482, Q04206, P01375, Q16539, P19838, P05231, P10145, P01584 |
| hsa04611 | Platelet activation | 0.0357721267299365 | P12931, P28482, Q16539 |
| hsa04380 | Osteoclast differentiation | 0.0000000076736284 | P28482, Q04206, P01375, Q16539, P19838, P37231, P01137, P01584 |
| hsa04370 | VEGF signaling pathway | 0.0003422715513945 | P12931, P35354, P28482, Q16539 |
| hsa04210 | Apoptosis | 0.0088428472979547 | Q04206, P01375, P19838 |
| hsa04151 | PI3K-Akt signaling pathway | 0.0430710019891760 | P28482, Q04206, P19838, P05231 |
| hsa04071 | Sphingolipid signaling pathway | 0.0001363008614475 | P28482, Q04206, P01375, Q16539, P19838 |
| hsa04066 | HIF-1 signaling pathway | 0.0000018439051577 | P05121, P28482, Q04206, P19838, P09601, P05231 |
| hsa04064 | NF-kappa B signaling pathway | 0.0000011282196303 | P35354, Q04206, P01375, P19838, P10145, P01584 |
| hsa04062 | Chemokine signaling pathway | 0.0000469464333268 | P12931, P13500, P28482, Q04206, P19838, P10145 |
| hsa04060 | Cytokine-cytokine receptor interaction | 0.0001674934921682 | P13500, P01375, P05231, P10145, P01137, P01584 |
| hsa04010 | MAPK signaling pathway | 0.0000136950065503 | P28482, Q04206, P01375, Q16539, P19838, P01137, P01584 |

**Supplementary Table 9 the topological parameters of target-pathway network**

| **name** | **Degree** | **name** | **Degree** |
| --- | --- | --- | --- |
| NFKB1 | 43 | hsa05168 | 6 |
| RELA | 43 | hsa05321 | 6 |
| MAPK1 | 35 | hsa05142 | 11 |
| TNF | 35 | hsa04668 | 10 |
| IL6 | 27 | hsa04621 | 9 |
| MAPK14 | 26 | hsa05152 | 9 |
| IL1B | 25 | hsa05161 | 9 |
| CXCL8 | 20 | hsa05164 | 9 |
| TGFB1 | 18 | hsa04380 | 8 |
| SRC | 13 | hsa04620 | 8 |
| CCL2 | 9 | hsa05133 | 8 |
| MMP9 | 5 | hsa05140 | 8 |
| MMP2 | 4 | hsa04010 | 7 |
| PTGS2 | 4 | hsa04932 | 7 |
| SERPINE1 | 2 | hsa05132 | 7 |
| HMOX1 | 1 | hsa05146 | 7 |
| PPARG | 1 | hsa05205 | 7 |
| hsa04370 | 4 | hsa04060 | 6 |
| hsa04623 | 4 | hsa04062 | 6 |
| hsa04722 | 4 | hsa04064 | 6 |
| hsa04912 | 4 | hsa04066 | 6 |
| hsa04915 | 4 | hsa05131 | 6 |
| hsa04931 | 4 | hsa05134 | 6 |
| hsa05162 | 4 | hsa05144 | 6 |
| hsa05203 | 4 | hsa05145 | 6 |
| hsa05212 | 4 | hsa05160 | 6 |
| hsa05220 | 4 | hsa05168 | 6 |
| hsa04210 | 3 | hsa05321 | 6 |
| hsa04611 | 3 | hsa05323 | 6 |
| hsa04640 | 3 | hsa04071 | 5 |
| hsa04662 | 3 | hsa04622 | 5 |
| hsa04664 | 3 | hsa04660 | 5 |
| hsa04920 | 3 | hsa04917 | 5 |
| hsa05143 | 3 | hsa05120 | 5 |
| hsa05169 | 3 | hsa05166 | 5 |
| hsa05215 | 3 | hsa05219 | 5 |
| hsa05221 | 3 | hsa04151 | 4 |
| hsa05332 | 3 | hsa05410 | 3 |

**Supplementary Table S10 The result of molecular docking**

| **Compound** | **Target** | **Binding Energy (kJ·mol^-1^)** |
| --- | --- | --- |
| rutaecarpine | NFKB1 | -7.20 |
| Stigmasterol | NFKB1 | -6.91 |
| beta-sitosterol | NFKB1 | -6.84 |
| rutaecarpine | MAPK1 | -6.66 |
| beta-sitosterol | MAPK1 | -5.87 |
| Stigmasterol | MAPK1 | -5.8 |
| kaempferol | NFKB1 | -5.74 |
| quercetin | MAPK1 | -5.43 |
| rutaecarpine | RELA | -5.43 |
| wogonin | NFKB1 | -5.40 |
| beta-sitosterol | RELA | -5.15 |
| rutaecarpine | TNF | -4.98 |
| Stigmasterol | RELA | -4.94 |
| wogonin | MAPK1 | -4.74 |
| baicalein | NFKB1 | -4.46 |
| kaempferol | MAPK1 | -4.37 |
| quercetin | NFKB1 | -4.32 |
| kaempferol | RELA | -4.29 |
| Stigmasterol | TNF | -4.01 |
| beta-sitosterol | TNF | -3.65 |
| wogonin | TNF | -3.52 |
| wogonin | RELA | -3.51 |
| kaempferol | TNF | -3.35 |
| quercetin | TNF | -3.22 |
| baicalein | MAPK1 | -3.09 |
| quercetin | RELA | -2.64 |
| baicalein | TNF | -1.38 |
| baicalein | RELA | -1.1 |
